# Space Charge Layer Evolution in All-Solid-State Batteries Probed via Operando Kelvin Probe Force Microscopy and Nuclear Reaction Analysis

**DOI:** 10.1021/acsnano.5c10125

**Published:** 2025-11-05

**Authors:** Chao Zhu, Shigeru Kobayashi, Yuki Sugisawa, Franjo Weber, Kun-Han Lin, Miho Kitamura, Koji Horiba, Hiroshi Kumigashira, Kazunori Nishio, Ryota Shimizu, Daiichiro Sekiba, Taro Hitosugi, Rüdiger Berger

**Affiliations:** † 28308 Max Planck Institute for Polymer Research, Ackermannweg 10, 55128 Mainz, Germany; ‡ Department of Chemistry, 13143 The University of Tokyo, Tokyo 113-0033, Japan; § Graduate School of Pure and Applied Sciences, 13121 University of Tsukuba, Tsukuba, Ibaraki 305-8573, Japan; ∥ 454209 National Institutes for Quantum Science and Technology (QST), Sendai 980-8579, Japan; ⊥ Institute of Multidisciplinary Research for Advanced Materials (IMRAM), 13101 Tohoku University, Sendai, Miyagi 980-8577, Japan; # School of Materials and Chemical Technology, Tokyo Institute of Technology, Tokyo 152-8552, Japan; ¶ Department of Chemical Engineering, 34881 National Tsing Hua University, Hsinchu 300044, Taiwan

**Keywords:** all-solid-state batteries, space charge layer, scanning force microscope, nuclear reaction analysis, Kelvin probe, nondestructive

## Abstract

The current controversies about the role of space charge layers hinder the development of better solid–solid interfaces and, thus, the improvement of solid-state batteries (ASSBs). To overcome this, we have combined high spatial resolution and nondestructive techniques, operando heterodyne Kelvin probe force microscopy (KPFM), and operando nuclear reaction analysis (NRA) to conduct a study of space charge layers in ASSBs. A model thin-film ASSB was fabricated from lithium (Li)|Li_3_PO_4_ (LPO)|LiCoO_2_ (LCO) for this study. This battery excels due to negligible interfacial defects and side reactions. For a working battery voltage range from 3.0 to 4.3 V vs Li/Li^+^, a space charge layer mainly exists at the LPO|LCO interface. This space charge layer with a width <50 nm arises from the redistribution of Li-ions at the interface. We clarified controversial views on the role of space charge layers in ASSBs by quantitatively determining the interfacial space charge layer resistance and found a maximum value between 18.4 and 19.1 Ω cm^2^ at 4.3 V vs Li/Li^+^. The absolute value of interfacial resistance from space charge layer formation is much smaller compared with the bulk solid electrolyte resistance in the fabricated thin-film ASSB. By employing KPFM and NRA techniques in ASSB research, our knowledge of space charge layer evolution at the solid electrolyte electrode interface is more comprehensive, even beyond the investigation of space charge layers.

## Graphical abstract

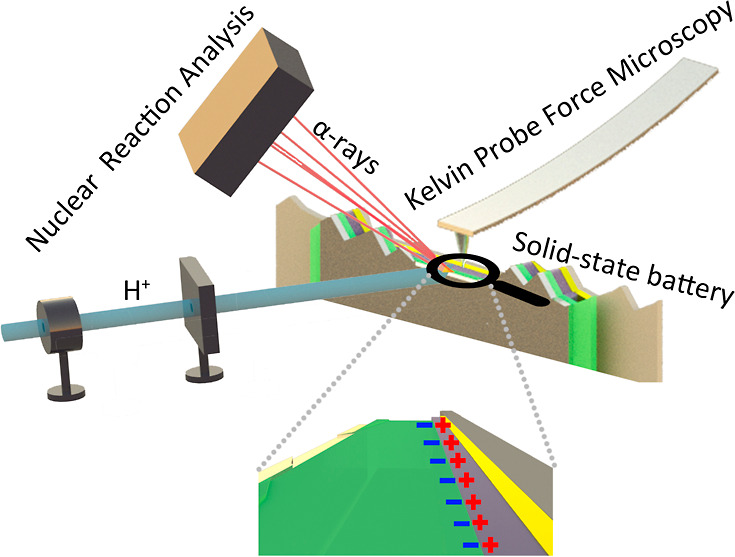

## 1 Introduction

All-solid-state batteries (ASSBs) are potential candidates for becoming the next-generation batteries. Compared with commercial lithium (Li) ion batteries, ASSBs have a higher energy density and a larger operation voltage window by utilizing metallic Li anodes.^1^ Using solid electrolytes instead of liquid electrolytes in ASSBs can improve battery safety.^2^ However, switching from a liquid to a solid electrolyte poses challenges at the solid electrolyte-to-electrode interface.^3–6^ In particular, a buildup of a space charge layer at the interface is likely to occur.^7,8^ Despite different methods of experimental and theoretical investigation, the evolution and effect of a space charge layer at the solid electrolyte-to-electrode interface in ASSBs are still unclear.

A space charge layer forms at the interface due to a redistribution of charge carriers near the interface, which is caused by a different chemical potential of Li in the electrode and the solid electrolyte.^9,10^ Contradictory conclusions^11^ on space charge layers are found in many studies using electrochemical impedance spectroscopy (EIS),^12^ density functional theory (DFT) calculation,^13,14^ in situ differential phase-contrast scanning transmission electron microscopy (DPC-STEM),^15^ two-dimensional nuclear magnetic resonance (NMR),^10^ and electron holography (EH).^16^ For example, DFT calculations of a Li_3_PS_4_ to LiCoO_2_ (LCO) interface predict Li-ion accumulation on the Li_3_PS_4_ side.^17^ In contrast, calculations based on the classical space charge layer model suggest that a higher Li-ion concentration should be present on the LCO side.^18^ Quantitative EH reveals a thickness of over 1 μm for a space charge layer at a Li_1+*x*+*y*_Al_*y*_Ti_2–*y*_Si_*x*_P_3–*x*_O_12_ to LCO interface.^11^ This thickness is significantly greater than the nm-thickness of a space charge layer estimated by the Poisson–Boltzmann equation based on the Gouy–Chapmann theory.^19^ Moreover, it remains uncertain how the low electronic conductivity of solid electrolytes contributes to the charge redistribution at interfaces in ASSBs. Electronic conductivity may influence the formation of space charge layers due to the possible internal polarization of solid electrolytes even in the electrochemical equilibrium state.^13,20^

In addition, the effect of the space charge layers on the performance of ASSBs is controversial. A space charge layer was widely thought to seriously hinder Li-ion transport across electrolyte-to-electrode interfaces. Thus, materials and interfaces need to be designed to eliminate an additional space charge layer resistance.^15,21,22^ However, Haruta et al. and Ohnishi and Takada recently mentioned that the electrical resistance originating from a space charge layer could be very small for clean and well-defined solid electrolyte-to-electrode interfaces, which are fabricated in a vacuum.^23,24^ In addition, it is unclear how the space charge layer depends on the state of charge (SOC). The above controversial statements motivated us to quantitatively investigate the evolution of the voltage-dependent space charge layer.

The main challenges for space charge layer investigation include a lack of suitable high spatial resolution and nondestructive detection techniques, which allow us to probe internal buried interfaces. Specifically, techniques need to be capable of an operando measurement that is able to characterize the properties under different battery SOCs. Commonly used EIS and NMR methods have low spatial resolution and are unable to resolve the impact that occurs at different interfaces.^10,25^ In contrast, high spatial resolution electron microscope techniques are often applied post-mortem and furthermore damage battery materials during measurements.^26^ Here, we overcome these limitations by developing nondestructive operando heterodyne Kelvin probe force microscopy (KPFM) and operando nuclear reaction analysis (NRA). Combining both techniques and in situ EIS enables us to quantitatively observe and analyze the origin and evolution of a space charge layer at solid electrolyte-to-electrode interfaces.

The investigation of space charge layers at solid electrolyte-to-electrode interfaces is also hampered by undefined contacts and possible side reactions at interfaces in batteries.^27,28^ Nowadays, solid electrolytes, such as sulfide-based, reach remarkable ionic conductivities of 10^–3^ S cm^–1^. However, they undergo interfacial reactions with electrode materials during cycling.^7^ Consequently, the space charge layer shifts to the interface between the electrode and the reaction-formed interphase. Although Li_3_PO_4_ (LPO) exhibits a lower conductivity of 10^–7^ to 10^–6^ S cm^–1^, it offers an important advantage: its conductivity closely matches that of the interphases formed in sulfide-based systems,^29^ making it a more representative platform for studying realistic space charge layer behavior in ASSBs. Furthermore, fabricated Li|LPO and LPO|LCO interfaces are stable and cause only negligible chemical side reactions (interfaces in the cell are indicated with a “|” symbol).^30^ In particular, the interfaces in the thin-film battery are well-defined and fabricated in close physical contact.^23,29,31^ Therefore, Li|LPO|LCO acts as a representative thin-film battery and a role model system for the investigation of space charge layers.

Here, we report that a space charge layer mainly exists at the LPO|LCO interface in a Li|LPO|LCO thin-film battery. During charging, the space charge layer evolves: Li-ions gradually accumulate on the LCO side, while they deplete on the LPO side close to the LCO|LPO interface. Both the potential change across the space charge layer and the corresponding resistance at the LPO|LCO interface increase monotonically for battery voltages increasing from 3.85 to 4.3 V vs Li/Li^+^. The maximum space charge layer resistance in the Li|LPO|LCO model thin-film battery is around 18–19 Ω cm^2^ at 4.3 V vs Li/Li^+^.

## 2 Results and Discussion

### 2.1 Characterization of a Thin-Film Battery

The structure of the role model thin-film battery is Li|LPO|LCO|Au|Al_2_O_3_ (Figure 1a and Supplementary Figure 1). Li, LPO, and LCO represent the most typical negative electrode, solid electrolyte, and positive electrode materials, respectively. Au is the current collector, and Al_2_O_3_ is the substrate. There are two well-defined solid-electrolyte|electrode interfaces: Li|LPO and LPO|LCO; both interfaces exhibit a sparse number of physical defects and only negligible chemical side reactions, due to our well-defined deposition methods.^23,24,29–31^

**Figure 1:**
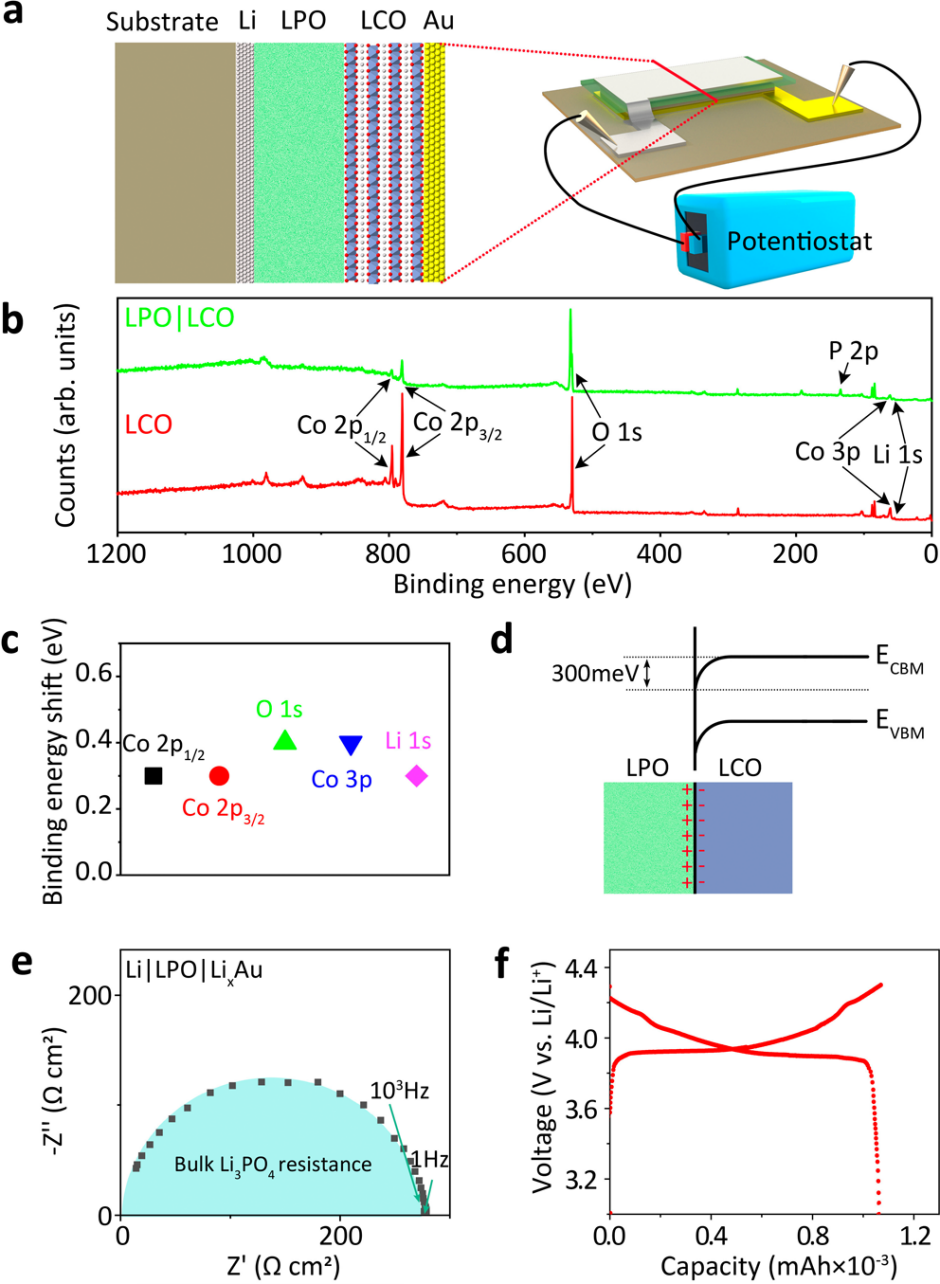
(a) Schematic diagram of a Li|LPO|LCO model thin-film battery device. (b) SXPS spectra of pure LCO and LCO coated with a thin LPO film. (c) Different core levels’ binding energy shift on LCO after LPO deposition. (d) Energy band diagram and schematic of charge redistribution at the LPO|LCO interface. (e) Nyquist plot of a Li|LPO|Li_*x*_Au cell. (f) Charging–discharging curves with a current density of 0.1 mA cm^–2^ of a Li|LPO|LCO thin-film battery.

At the beginning of our research, we investigated the pristine state of space charge layers for newly fabricated LPO|LCO and Li|LPO interfaces in two separate samples. Both interfaces corresponded to a battery charge state with an open circuit voltage (OCV) of around 2 V vs Li/Li^+^.^32^ For the LPO|LCO interface, we performed synchrotron X-ray photoelectron spectroscopy (SXPS) on a pristine LCO surface and on an LCO film that was coated with an ≈2 nm thick LPO layer (Figure 1b). Compared with the pristine LCO film, the P 2p signal indicated the deposition of LPO on LCO for the LPO|LCO stack (Figure 1b and Supplementary Figure 2). The Co 2p and Co 3p signals were present, which proves that SXPS still probes the LPO|LCO interface. The Co 2p_1/2_, Co 2p_3/2_, Co 3p, O 1s, and Li 1s emissions shifted by 0.3–0.4 eV toward higher binding energy in LCO after LPO deposition (Figure 1c and Supplementary Figure 3), while the reference carbon signal did not shift. XPS measures the binding energy relative to the vacuum level; therefore, an increase in binding energy reflects a downward shift of all electronic energy levels, including core levels, with respect to the vacuum level.^33^ This observation indicates the presence of downward band bending on the LCO side at the interface. Such a downward bending can be attributed to the accumulation of Li vacancies having a negative charge on the LCO side close to the interface. Conversely, Li ions with a positive charge accumulate on the LPO side adjacent to the interface. This interfacial charge accumulation leads to the formation of a space charge layer (Figure 1d).^34^ These findings are consistent with previous reports on LiPON-coated LCO systems.^21^

To investigate the Li|LPO interface, we prepared a Li|LPO|Li_*x*_Au cell and performed EIS measurements. We chose this cell structure to circumvent Li surface oxidation that occurs before LPO deposition, even in an inert environment. The fabrication of a Au underlayer ensures a pristine Li|LPO interface for analysis. The corresponding Nyquist plot (Figure 1e) shows only one semicircle in the high-frequency range (>10^3^ Hz). This semicircle corresponds to the bulk resistance of the solid electrolyte LPO.^35^ The LPO layer ionic conductivity estimated from the Nyquist plot corresponds to 8.7 × 10^–7^ S cm^–1^, which matches well with previously reported values.^36,37^ As we did not observe other semicircles in the Nyquist plot, the interfacial resistance, including space charge layer resistance and charge transfer resistance, at the Li|LPO interface was negligible in the pristine state. The electrode was made from Li–metal and did not change with battery SOC. Therefore, the charge transfer resistance at the Li|LPO interface was negligible regardless of the battery SOC, even though LPO could be thermodynamically unstable with Li.^5^

However, the following questions remain: Do space charge layers evolve in magnitude and polarization direction for different SOCs? Can the space charge layer contribution at different interfaces be separated? Can we explore and clarify the origin of the space charge layer evolution at the battery interfaces? Can we quantitatively evaluate the effect of the space charge layers for ASSBs? To answer these questions, we need to charge a battery to different SOCs to explore the evolution of space charge layers in the voltage range from 3.0 to 4.3 V vs Li/Li^+^, which is a widely used voltage range for operating LCO-based batteries to avoid interfacial side reaction and decomposition.^38^ Charge–discharge curves (Figure 1f) of the Li|LPO|LCO thin-film battery show the typical plateaus of Li-ions extracting from (charging) and inserting into LCO (discharging). The specific discharge capacity of LCO in the battery is around 125 mAh g^–1^. Cyclic voltammetry (CV) experiments (Supplementary Figure 4) support the above conclusion. More importantly, over 99.5% of the battery capacity remained after 80 cycles (Supplementary Figure 5). Thus, the LCO and the internal interfaces in the thin-film battery are stable during cycling. This stability indicates that both interfaces in our thin-film battery remain in close contact and do not exhibit significant side reactions during battery cycling.

### 2.2 KPFM Measurements of a Thin Battery Cross-Section

We used operando heterodyne KPFM to investigate the contact potential difference (CPD) distribution on the whole thin-film battery cross-section.^39–41^ We began by cleaving a thin-film battery. The exposed cross-section of the cleaved battery was too rough to apply heterodyne KPFM owing to signal crosstalk between the topography and CPD.^42–44^ Therefore, we applied argon-ion milling from the side to polish the exposed cross-section (Figure 2a). Using heterodyne KPFM, we mapped the CPD of the polished cross-section at different battery SOCs, controlled by an external potentiostat (Figure 2b).

**Figure 2:**
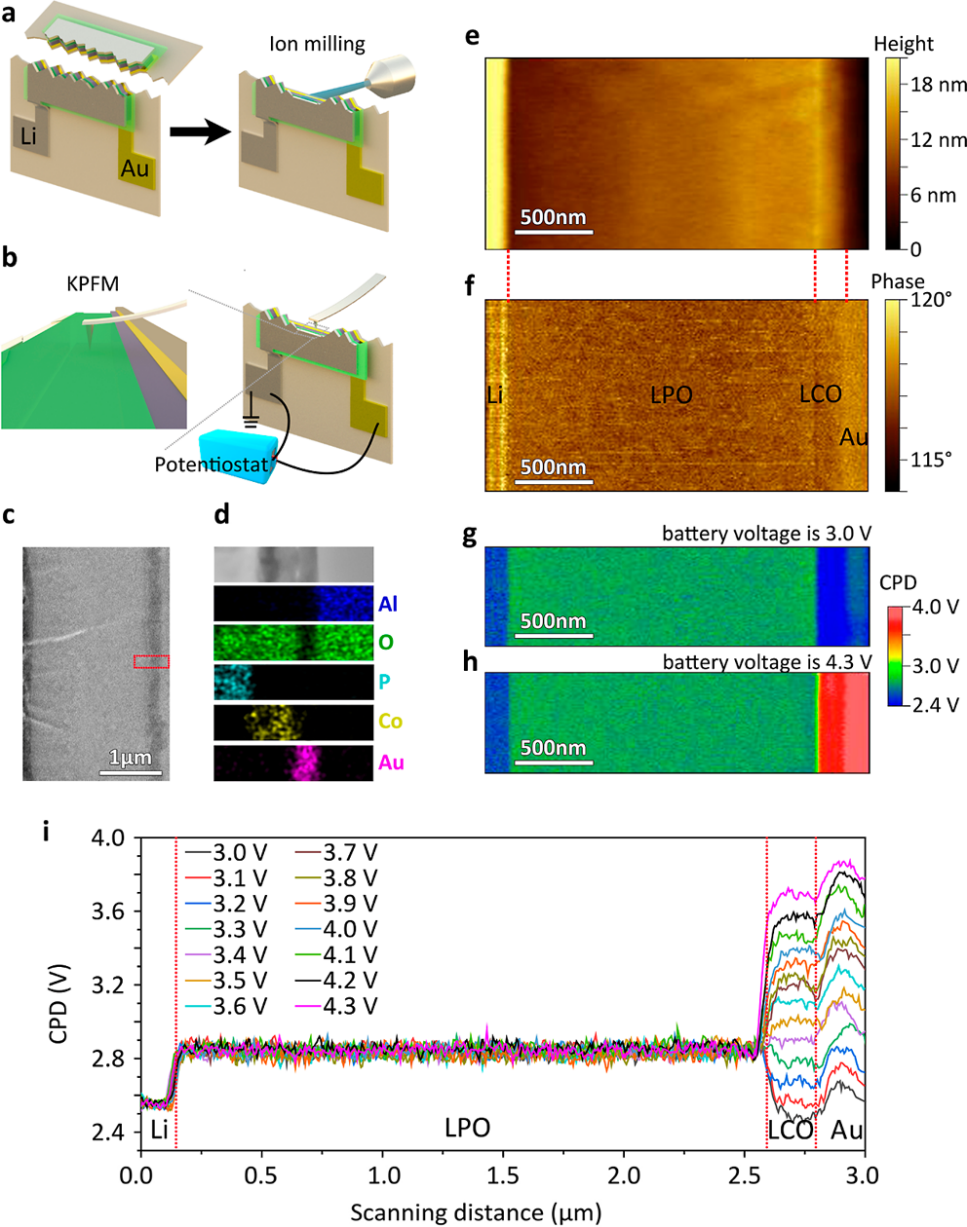
(a) Schematic diagram of cleaving and polishing a Li|LPO|LCO thin-film battery to prepare a cross-section. (b) Schematic diagram of operando heterodyne KPFM on a polished thin-film battery cross-section. (c) Scanning electron microscopy (SEM) of the polished cross-section. (d) EDX map of the region marked with a red square in (c). (e) Topography and (f) phase map of the polished Li|LPO|LCO thin-film battery cross-section. (g,h) CPD maps of the polished Li|LPO|LCO thin-film battery cross-section at battery voltages of 3.0 and 4.3 V vs Li/Li^+^, respectively. (i) Corresponding line profiles of the CPD at 14 different battery voltages.

Scanning electron microscopy (SEM) characterization on the polished cross-section revealed that interfaces in the thin-film battery were sharp and straight (Figure 2c). The elemental distribution map acquired from energy-dispersive X-ray analysis (EDX) confirmed that polishing did not mix the elements at the interfaces (Figure 2d). In addition, no change in the electrochemical properties was observed after polishing (Supplementary Figure 6).

The scanning force microscopy topography mapping (Figure 2e) and phase mapping (Figure 2f) of the polished cross-section acquired from the heterodyne KPFM measurements clearly displayed different layers. As expected, the thicknesses of the LPO layer and the LCO layer were 2500 nm and 200 nm, respectively. After polishing, the surface roughness of each layer was <3 nm, which is smooth enough to not affect KPFM signal collection on each layer.

The CPD maps of the whole battery cross-section at 3.0 and 4.3 V vs Li/Li^+^ showed changes only at the LCO and the Au layers, while the CPD of the Li and LPO layers remained constant (Figure 2g and h). Additional measurements along the whole battery cross-section in the voltage range from 3.0 to 4.3 V vs Li/Li^+^ in steps of 0.1 V confirmed this finding (Figure 2i). The CPD evolution of the LCO and the Au layers was also observed within one measurement, where the CPD was mapped continuously while charging the battery with a low external current (Supplementary Figure 7).

Li is a metal and was grounded. The CPD value of the Li surface remained constant at ≈2.55 V. The measured CPD value was consistent with the work function difference between the Pt–Ir-coated tip (Φ_Pt/Ir_ ≈ 5–5.93 V) and that of Li (Φ_Li_ ≈ 2.9 V). The constant CPD value of the Li and LPO layers indicated that the Galvani potential φ of both layers was fixed in the entire operating voltage range of the battery.^45,46^ The constant CPD difference between LCO and Au indicates no lithiation in the Au layer and no Li ion exchange between LCO and Au. Thus, the space charge layer resistance in the Li|LPO|LCO thin-film battery mainly arises at the LPO|LCO interface. To quantitatively trace CPD change on the LCO layer with higher resolution, we focused on the LPO|LCO interface by decreasing the scan area of the heterodyne KPFM measurements to 500 nm × 100 nm.

### 2.3 KPFM Measurements at the LPO|LCO Interface

In contrast to the constant CPD value of the LPO layer, the CPD value of the LCO layer continuously increased with increasing battery voltage, which is represented by the change from a blue to red color in the CPD maps (Figure 3a). Figure 3b shows the line profiles of the CPD values across the LPO and the LCO layers extracted from the CPD maps of Figure 3a. This evolution of the measured CPD with battery voltage is fully reversible. Even after 80 charge–discharge cycles, the CPD maps and corresponding CPD line profiles at different battery voltages (Supplementary Figure 8) are identical compared to those of the first cycle. Thus, the CPD change is caused by mobile charge carriers but not by interfacial irreversible side reactions.

**Figure 3:**
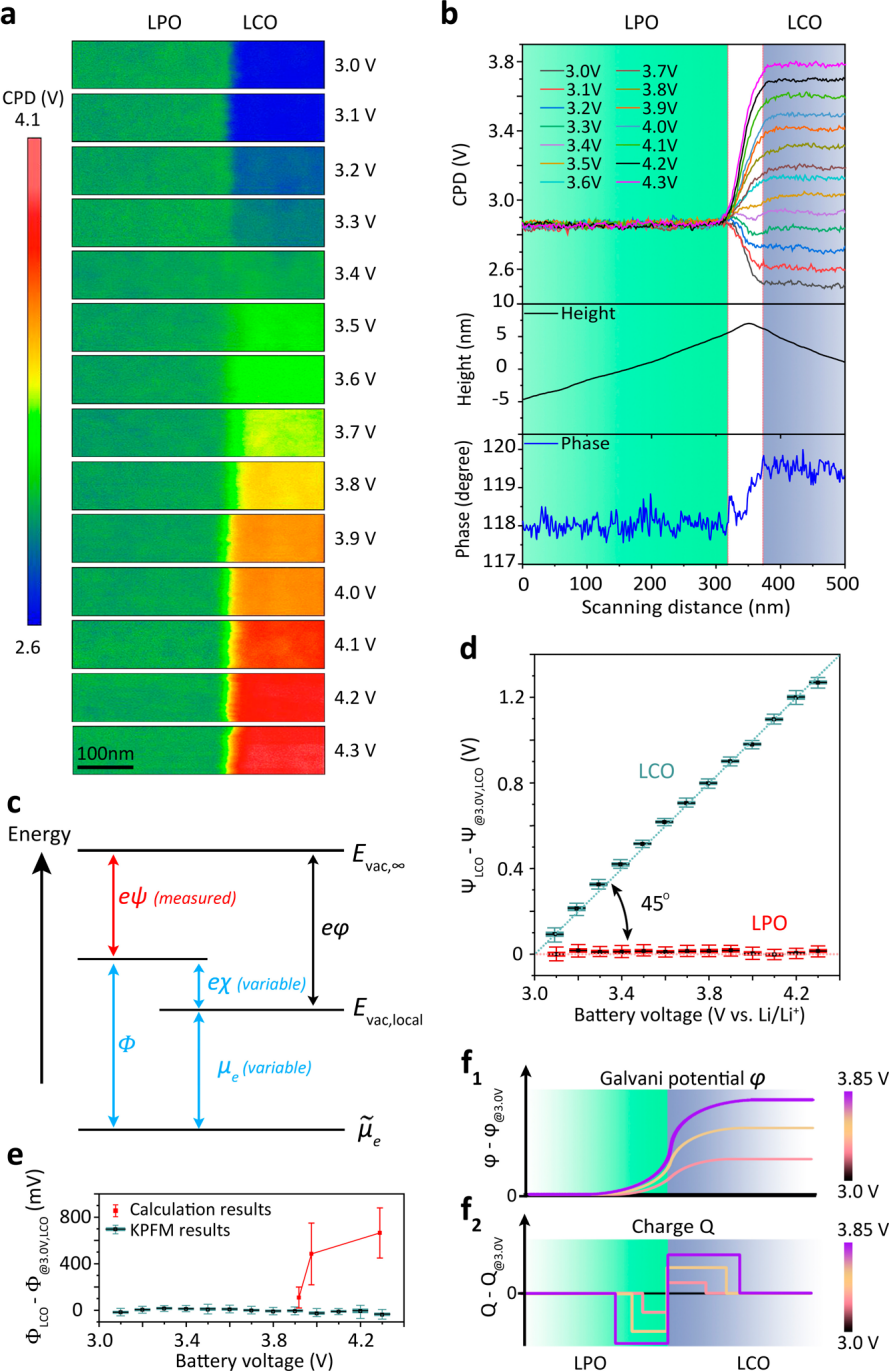
(a) CPD maps of the LPO and LCO layers at their interface at different battery voltages. (b) CPD line profiles at different battery voltages extracted from the CPD maps provided in (a) and the corresponding line profiles of the measured height and phase across the LPO and LCO layers. (c) Schematic energy diagram of the Volta potential (ψ), surface potential (χ), Galvani potential (φ), work function (Φ) chemical potential of electrons (μ_*e*_), and electrochemical potential of electrons ( ${\mathrm{\mu ̃}}_{e}$ ). (d) ψ mean value changes calculated from all the pixels on the LCO and LPO layers’ surface at different battery voltages compared to 3.0 V vs Li/Li^+^. In this analysis, we excluded the LPO|LCO transition region. (e) Comparison of work function Φ changes of the LCO layer under different battery voltages relative to 3.0 V vs Li/Li^+^, acquired from DFT calculations and KPFM measurements, respectively. (f_1_–f_2_) Schematic diagrams of the Galvani potential change, charge concentration changes at the LPO|LCO interface. All changes are drawn relative to a reference voltage of 3.0 V vs Li/Li^+^ and are valid for a battery voltage range of 3.0 V–3.85 V vs Li/Li^+^.

The CPD profiles at different battery voltages indicated a transition region between the LPO and the LCO layers with a width of around 50 nm (marked with a white background in Figure 3b). This width represents an upper limit for the true space charge layer thickness. The displayed transition region is determined by the intrinsic spatial resolution of the KPFM technique. The resolution is fundamentally limited by the probe geometry, e.g., the tip radius with several tens of nanometers and long-range electrostatic forces. Additionally, the local sample topography at the interface, i.e., a characteristic kink at the interface due to the different polishing rate on the LPO and LCO layers (Figure 3b and Supplementary Figure 9), further decreases the measurement resolution. Thus, the actual space charge layer at the LPO|LCO interface is likely narrower than 50 nm. The CPD and phase maps further revealed that no side reaction took place at the LPO|LCO interface, as no interphase was observed. For comparison, we prepared a battery where the LCO surface was damaged due to a higher deposition rate of the LPO. This led to an interphase formation between the LPO and LCO layers owing to an interfacial side reaction.^28^ In this case, heterodyne KPFM detected the existence of the additional interphase with a thickness of around 30 nm (Supplementary Figure 10). We note that this thickness value represents a spatially averaged signal. The measurement demonstrates the technique’s sensitivity to a gradient in potential. The true interphase thickness, expected to be in a nanometer scale, remains below the instrument’s resolution limit for measuring the CPD.

In our heterodyne KPFM measurements, from a physical chemistry point of view, the measured CPD change (ΔCPD) equals the Volta potential change (Δψ),^45^ which we outline in a schematic energy diagram (Figure 3c)

1 $$\Delta CPD=\Delta \psi =\Delta \varphi -\Delta \chi =\frac{\Delta {\mathrm{\mu ̃}}_{e}-\Delta \Phi }{e}$$

where Δφ is the change of Galvani potential (or inner potential), Δχ is the change of surface potential, $\Delta {\mathrm{\mu ̃}}_{e}$ is the change of the electrochemical potential of electrons, ΔΦ is the change of the work function, and *e* is the charge of electrons. The following correlations are valid (Figure 3c)

2 $$\Phi =\mu_{e}+e\chi$$

3 $${\mathrm{\mu ̃}}_{e}=\mu_{e}+e\varphi$$

where μ_*e*_ is the chemical potential of electrons.

We quantitatively analyzed the ΔCPD (Δψ) on the LPO and LCO layers at different battery voltages relative to a reference ψ_@3.0V_ measured at 3.0 V vs Li/Li^+^ (Figure 3d). Our measurements showed that Δψ of the LPO layer (Δψ_LPO_) remains constant at 0. We concluded from eq 1 that the work function of LPO (Φ_LPO_) and the electrochemical potential of electrons ( ${\mathrm{\mu ̃}}_{e,LPO}$ ) do not depend on battery voltage. In contrast, Δψ of the LCO layer (Δψ_LCO_) is directly proportional to the change in the battery voltage, with a slope of 1. This proportionality indicates that Δψ_LCO_ only results from the change in the electrochemical potential of electrons in LCO ( $\Delta {\mathrm{\mu ̃}}_{e,LCO}$ ). The change of battery voltage measured by a potentiostat equals $\Delta {\mathrm{\mu ̃}}_{e,LCO}/e$ when the Li electrode is grounded in our thin-film battery.^47^ Therefore, the work function change of LCO (ΔΦ_LCO_) is constant at 0 (Figure 3e).

At battery voltages of <3.85 V vs Li/Li^+^, few Li-ions deintercalate from bulk LCO, and a constant Φ_LCO_ can be expected. However, at battery voltages of >3.85 V vs Li/Li^+^, Li-ions extracted from LCO and Φ_LCO_ should no longer be constant as the Li content decreases significantly. We verified this result by performing operando heterodyne KPFM measurements on a thin-film battery where the LCO layer is grounded (Supplementary Figure 11 and Supplementary Note 1) and on a thin-film battery with a freshly cleaved LCO surface that has not undergone any polishing process (Supplementary Figure 12 and Supplementary Note 2). A constant Φ_LCO_ was also observed by Fuller et al. in a recent KPFM experiment.^48^ Differently, DFT calculations revealed an increasing Φ_LCO_ for a battery voltage of >3.85 V vs Li/Li^+^ (Figure 3d and supplementary Figure 13a). We attribute the discrepancy between the calculated and experimental results to the absence of Li ion extraction from the surface of LCO at voltages exceeding 3.85 V vs Li/Li^+^ (supplementary Figures 13b and 14, Supplementary Note 3). Possibly, the energy barrier for delithiation from the surface is much higher compared to that of bulk LCO, due, for example, to the presence of a surface dipole.^49^

In summary, heterodyne KPFM only provided information on the space charge layer evolution at the LPO|LCO interface in the battery voltage range from 3.0 to 3.85 V vs Li/Li^+^. In this voltage range, we found that ψ_LPO_ and Φ_LPO_ are constant, leading to a constant φ_LPO_. Thus φ_LPO_-φ_@3V,LPO_ is 0 (Figure 3f_1_). For the LCO layer, a constant Φ_LCO_ indicates a constant μ_e,LCO_ and χ_LCO_ based on eq 2. With an increase in the battery voltage, ψ_LCO_ increases. Based on eq 1, we concluded that φ_LCO_ increases relative to the reference φ_@3V,LCO_ (Figure 3f_1_). This increase in φ_LCO_ results from an increasing built-in electrical field at the LPO|LCO interface (Supplementary Figure 15). The increasing built-in electrical field originates from a gradual accumulation of negative charges at the LPO side close to the interface, while positive charges gradually accumulate at the LCO side close to the interface (Figure 3f_2_). Furthermore, considering the changes in the electronic energy band and Fermi level of LPO relative to LCO at different voltages (Supplementary Figure 16), we can conclude that the positive charges are mainly Li^+^ ions, while the negative charges are Li vacancies (Supplementary Note 4).^10,13,50^

To verify the conclusion based on operando heterodyne KPFM and to further study space charge layer evolution in a battery voltage range higher than 3.85 V vs Li/Li^+^, we used operando NRA to directly measure the Li concentration evolution at the LPO|LCO interface.

### 2.4 NRA Analysis and Theoretical Space Charge Layer Calculation

We recently applied an operando NRA technique to probe the depth profile of the ^7^Li density in thin-film batteries with a depth resolution of ∼50 nm. An incidental H^+^ ion beam with an energy of 1 MeV is directed toward the sample, resulting in a nuclear reaction between the H^+^ and ^7^Li. The ^7^Li­(p, α)^4^He reaction results in the production of α-rays, which are subsequently detected and quantified for the measurement of depth-dependent interactions in materials (Figure 4a). Figure 4b shows the NRA spectra of the battery obtained at a voltage of 3.0 V vs Li/Li^+^. Our multilayer fitting model of the NRA spectra considered the Li content in the Li layer (gray), LPO layer (green), and LCO layer (blue) in the thin-film battery. The NRA spectra at 3.0 V vs Li/Li^+^ show good repeatability at different measurements for the same battery (Supplementary Figure 17a), which proves the reliability of NRA measurements on our system. Additional NRA spectra at different SOCs and corresponding fitting results are provided in Supplementary Figure 17b.

**Figure 4:**
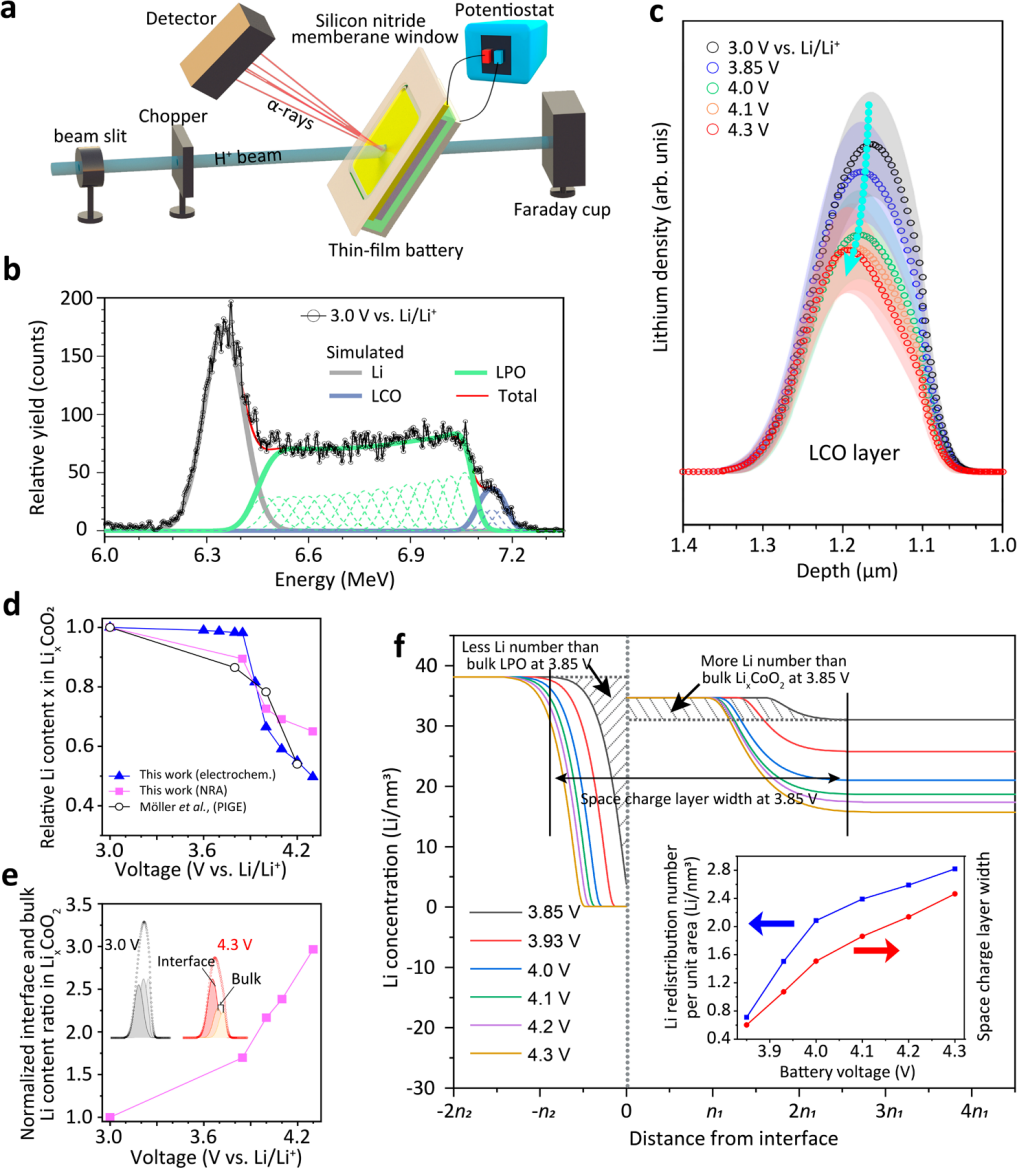
(a) Schematic diagram of the operando NRA experiment of a thin-film battery. (b) NRA signal vs scattered α-particle energy at 3.0 V vs Li/Li^+^. An increasingly scattered α-particle energy leads to a signal from a deeper volume of the battery. The fits of the components, Li, LCO, and LPO, are provided. (c) The Li content obtained from the fits at the LCO layer at different battery voltages. (d) Comparison of LCO bulk Li content change with battery voltage acquired from our NRA results and reported using the PIGE technique.^51^ (e) Normalized Li content ratio evolution with battery voltage in LCO close to the LPO|LCO interface and in LCO far from the LPO|LCO interface. The ratio at a voltage of 3.0 V vs Li/Li^+^ is the reference used for normalization. (f) Simulation of the Li-ion concentration profile of the space charge layer at the LPO|LCO interface with battery voltage. The area of the hatched region in the LPO layer is the number of Li vacancies per unit area at 3.85 V, while the area of the hatched region in the LCO layer is the number of excess Li-ions per unit area compared to bulk LCO at 3.85 V vs Li/Li^+^. The inset figure shows the evolution of the number of Li-ions per unit area redistributed from LPO to LCO to form a space charge layer and the width of the space charge layer at the LPO|LCO interface as a function of the battery voltage.

In the LCO layer, the fitting results showed that the overall signal of Li content significantly decreased when charging the battery from 3.0 to 4.3 V vs Li/Li^+^ (Figure 4c). In contrast, the depth profile of other heavier elements than Li measured by Rutherford backscattering (RBS) in the battery did not change as expected (Supplementary Figure 18). We plotted the changes of the Li content in the LCO layer with increasing battery voltage, as shown in Figure 4d. These changes are in good agreement with the results calculated from the charge–discharge curve and the results of particle-induced gamma-ray emission analysis (PIGE).^51^ We attribute the small decline in Li content in the bulk LCO for increasing voltages <3.85 V vs Li/Li^+^ to the inherent nature of LCO pristine defects formed from battery fabrication. In addition, we cannot exclude that noise and vibration during the recording of the NRA signal lead to such a decrease as well.

More interestingly, the peak center of the Li content in LCO shifted toward the LPO|LCO interface when increasing the battery voltage from 3.0 to 4.3 V vs Li/Li^+^ (Figure 4c). To determine the peak center shift of the Li content more accurately, we fitted three Gaussian curves to the NRA signal of Li in the LCO layer (Figure 4e). One Gaussian curve corresponds to the Li content in the LCO layer close to the LPO|LCO interface (Υ_LCO,interface_), while the other two correspond to the Li content in the LCO layer far from the LPO|LCO interface (Υ_LCO,bulk_) (Figure 4e and Supplementary Figure 19). We normalized the ratio Υ_LCO,interface_/Υ_LCO,bulk_ as 1 at 3.0 V vs Li/Li^+^. The ratio Υ_LCO,interface_/Υ_LCO,bulk_ continuously increased with increasing battery voltage to ∼2.97 at 4.3 V vs Li/Li^+^ (Figure 4e). This finding verifies the conclusion from KPFM measurements that Li-ions gradually accumulate at the LCO side at the LPO|LCO interface at a battery voltage of <3.85 V vs Li/Li^+^. Furthermore, operando NRA proves that Li-ions continued to accumulate on the LCO side for battery voltages higher than 3.85 V vs Li/Li^+^. Thus, φ_LCO_ increases relative to φ_LPO_ with increasing battery voltage in the whole battery operating voltage window. Our operando NRA measurements did not reveal a decrease in Li content in LPO close to the interface with increasing voltage (Supplementary Figure 20). Possibly, the Li depletion width on the LPO side is too narrow to be detected with NRA because of different ionic conductivities and permittivities in LPO compared with LCO.^18,32,52^ Additionally, the amount of depleted Li at the LPO side is much smaller compared to the bulk LPO and thus cannot be detected.

At voltages >3.85 V vs Li/Li^+^, the electrochemical potential of Li-ions in the LPO and LCO layers always equals to each other in the equilibrium state because Li-ions can deintercalate from LCO with increasing battery voltage in this voltage range. Then the space charge layer calculation model proposed by de Klerk et al. applies.^53^ Using this model, we calculated the Li concentration profile at the LPO|LCO interface (Figure 4f). The model is based on a solid solution that obeys mass conservation to describe the relationship between the Li-ion chemical potential and its distance to the interface. The chemical potential of Li-ions is determined by its concentration (Supplementary Figure 21, Tables 1 and 2, and Supplementary Note 5). An equilibrium potential of 0.7 V for LPO vs Li/Li^+^ was used in the calculations.^36^ We calculated space charge layers at the LPO|LCO interface for different battery voltages of 3.85, 3.93, 4.0, 4.1, 4.2, and 4.3 V vs Li/Li^+^. The calculation takes into consideration the lattice constant, the local dielectric constant, and the energetic difference between lattice sites occupied by Li ions and empty sites in LPO and LCO. For simplicity, these constants were summarized in the terms *n*_1_ for LCO and *n*_2_ for LPO in Figure 4f. The calculation results show that Li accumulation on the LCO side (Li depletion on the LPO side) and the width of the space charge layer increased with battery voltage, which matches well with our operando NRA results.

### 2.5 Space Charge Layer Evolution

We provide a summary of our findings: (1) A space charge layer mainly exists and plays a role at the LPO|LCO interface in our thin-film battery. (2) The space charge layer width is <50 nm. (3) The space charge layer at the LPO|LCO interface evolves with Li-ion accumulation on the LCO side and Li-ion depletion on the LPO side with increasing battery voltages. (4) The Galvani potential φ_LCO_ increases relative to φ_LPO_ for increasing battery voltages.

The electrochemical potential of Li^+^ ( ${\mathrm{\mu ̃}}_{{Li}^{+}}$ ) is the sum of the chemical potential of Li-ions (μ_Li^+^_) and φ. The chemical potential μ_Li^+^_ in bulk LPO and LCO is constant for voltages of <3.85 V vs Li/Li^+^ as no Li-ions intercalate into/deintercalate from LCO. Therefore, we conclude: (5) ${\mathrm{\mu ̃}}_{{Li}^{+}}$ in LCO continuously increases with battery voltage until it equals that in LPO for a battery voltage of >3.85 V vs Li/Li^+^, where Li-ions begin to extract from or insert into LCO. We have observed a continuous CPD value increase in the LCO layer, which corresponds to the battery voltage increase from 2.0 to 3.0 V vs Li/Li^+^. In this voltage range, the CPD value of the LPO layer remains constant (Supplementary Figure 22). Therefore, all five findings can be extrapolated down to 2.0 V vs Li/Li^+^, which is the OCV of our battery.

The next step is to determine the space charge layer evolution for different battery SOCs. We sketched six states (Figure 5a–f): Before battery fabrication, i.e., when the LPO was not deposited on LCO, potentials are constant in both layers (Figure 5a). Next, we brought the LPO and the LCO layers into contact, i.e., a newly fabricated thin-film battery. In this case, the ${\mathrm{\mu ̃}}_{{Li}^{+}}$ difference between LPO and LCO causes Li-ions in LPO to move toward the LCO interface. However, these Li-ions cannot insert into the LCO at this stage. As a result, Li-ions accumulated in LPO at the LCO interface. Due to charge neutrality, Li-ions in LCO move away from the interface and leave Li vacancies. These vacancies are negatively charged. This charge state was measured by SXPS (Figure 1d). At the same time, a built-in electrical field formed at the interface until the battery voltage turned to ∼2 V vs Li/Li^+^ and reached a stable state (Figure 5b). By charging the battery, Li-ions in the LPO move to the Li-electrode and are reduced into metallic Li due to the applied external bias. Thus, the Li-ion accumulation decreases at the LPO side close to the LPO|LCO interface. At the same time, to keep local charge neutrality, Li-ions in bulk LCO move to the interface but cannot be extracted from LCO at battery voltages of <3.85 V vs Li/Li^+^. As a result, the number of Li vacancies in the LCO close to the interface decreases. This means that the built-in electrical field at the LPO|LCO interface decreases. Thus, both ${\mathrm{\mu ̃}}_{{Li}^{+}}$ and φ in bulk LCO increase compared to bulk LPO (Figure 5c). The charge capacity below 3.85 V vs Li/Li^+^ accounts only for 1.7% of the total charge capacity up to 4.3 V vs Li/Li^+^ (calculated from Figure 1f). This charge capacity is attributed to ion redistribution at the interface before bulk Li delithiation from LCO. By continually charging the battery, all accumulated charges at the LPO|LCO interface disappeared, and the space charge layer vanished. This battery voltage corresponds to *V*_no‑SCL_. At this voltage, the built-in electrical field at the interface disappears. Thus, φ in LPO and LCO across its interface stays constant (Figure 5d). Our above calculation on LPO|LCO interfacial space charge layer (Figure 4f) shows that *V*_noSCL_ should be <3.85 V vs Li/Li^+^. Then, by continually charging the battery, the space charge layer forms again at the LPO|LCO interface. Now the polarization direction reverses its direction, indicating that Li-ions accumulate (deplete) at the LCO (LPO) side. Accordingly, φ in LPO becomes larger than in LCO (Figure 5e). Until the battery voltage is >3.85 V vs Li/Li^+^, Li-ions can extract from LCO with increasing battery voltage. As Li-ions can now move across the LPO|LCO interface, ${\mathrm{\mu ̃}}_{{Li}^{+}}$ in LCO and LPO will equilibrate under OCV conditions. Given this, the Li-ion concentration in LCO decreases, which decreases μ_Li^+^_ of LCO. To maintain the ${\mathrm{\mu ̃}}_{{Li}^{+}}$ equilibrium between LCO and LPO, φ of LCO increases due to the accumulation of more Li-ions at the LCO side and more Li vacancies at the LPO side of the interface. This further strengthens the space charge layer at the LPO-LCO interface (Figure 5f).

**Figure 5:**
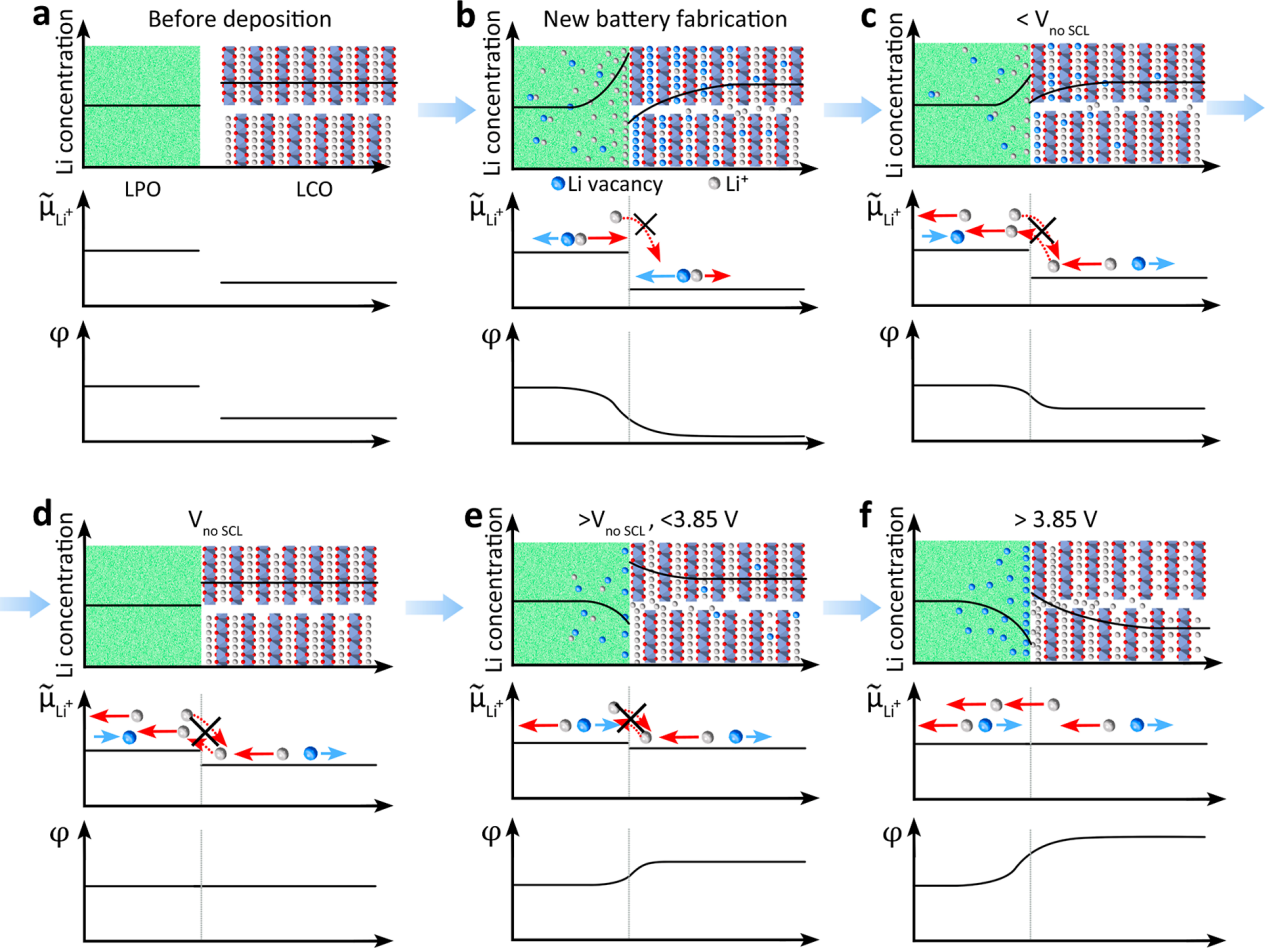
(a–f) Li-ion concentration, ${\mathrm{\mu ̃}}_{{Li}^{+}}$ and φ evolution in bulk LPO, LPO|LCO interfaces, and bulk LCO before battery assembly and at different battery voltages.

### 2.6 Calculation of the Space Charge Layer Resistance

In this section, we quantitatively evaluate the effect of the space charge layer based on the conclusions we got from operando KPFM/NRA characterizations and in situ EIS measurements. In the voltage range from 3.0 to 4.3 V vs Li/Li^+^ (Figure 6a,b) and in the high-frequency range (>10^3^ Hz) of the Nyquist plots, we observed the same semicircle as for Li|LPO|Li_*x*_Au cell results from the bulk resistance of the solid electrolyte LPO. All those semicircles recorded at different battery voltages overlap, indicating a constant bulk resistance of LPO.

**Figure 6:**
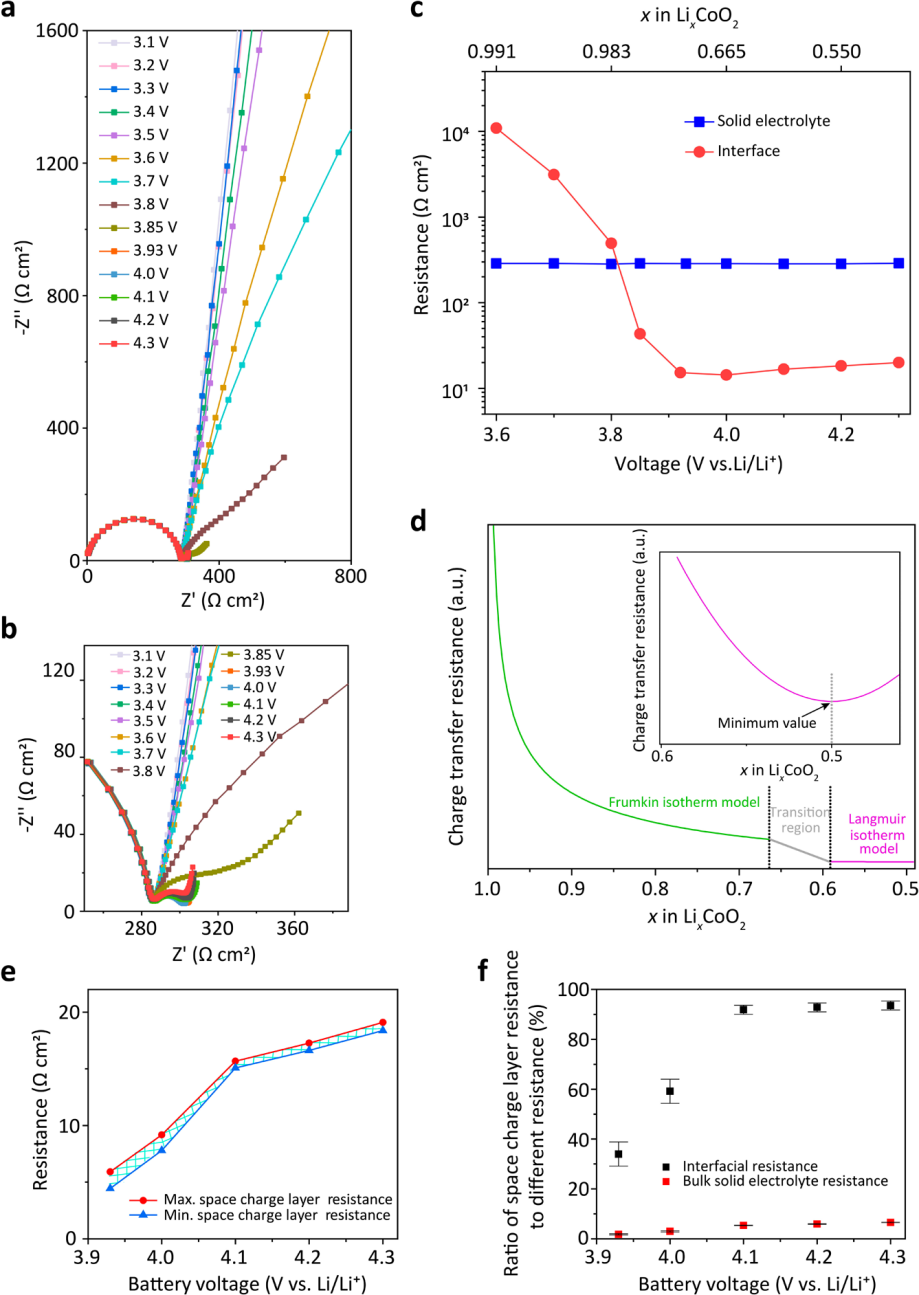
(a) Nyquist plots of a Li|LPO|LCO thin-film battery at different voltages. (b) Magnification of the Nyquist plots in the high- and midfrequency range in (a). (c) LPO bulk resistance and interfacial resistances extracted by fitting the data from (b). (d) Calculated LPO|LCO interfacial charge transfer resistance in dependence on the Li-ion concentration x in LCO. (e) Calculated space charge layer resistance at different battery voltages. (f) Ratio of space charge layer resistance to the whole interfacial resistance and bulk solid electrolyte resistance at different battery voltages.

The slope in the mid- and low-frequency range (<10^3^ Hz) of the Nyquist plot at voltages of <3.6 V vs Li/Li^+^ indicated that almost no redox reaction occurred in this voltage range. When the battery was charged to >3.6 V vs Li/Li^+^, another semicircle in the mid- and low-frequency range of the Nyquist plot gradually developed, which turned to a full semicircle at 3.85 V vs Li/Li^+^. Delithiation in bulk LCO began at a voltage around 3.85 V vs Li/Li^+^. We modeled the second semicircle in Nyquist plots with an equivalent circuit given by a resistor in series with a capacitor. Since there was no interphase formation at the LPO|LCO interface, this equivalent circuit describes the mid- and low-frequency range very well (Supplementary Figure 23a). We attribute the resistor in our model to the interfacial resistance.^23^ As a comparison for the thin-film battery with an additional interphase between the LPO and LCO layers detected with KFPM, the Nyquist plots in mid- and low-frequency ranges exhibit two semicircles (Supplementary Figure 23b). In this case, the additional semicircle indicated the formation of the interphase. The interfacial resistance decreased from around 10.8 kΩ cm^2^ at 3.6 V vs Li/Li^+^ to 14.3 Ω cm^2^ at 4.0 V vs Li/Li^+^ and then slightly increased to around 20.0 Ω cm^2^ at 4.3 V vs Li/Li^+^. The lowest interfacial resistance was at 4.0 V vs Li/Li^+^, which is close to the lowest value recently reported.^24^ By using the charge–discharge curves (Figure 1f) and the widely reported value of *x* ≈ 0.55 in Li_*x*_CoO_2_ at 4.2 V vs Li/Li^+^,^37,54^ we calculated that a voltage of 4.0 V vs Li/Li^+^ corresponds to a lithiation degree of *x* ≈ 0.665 in Li_*x*_CoO_2_ (Figure 6c). The evolution of interfacial resistance is reversible when decreasing the battery voltage (Supplementary Figure 24).

In our role model thin-film battery, both Li|LPO and LPO|LCO interfaces can be seen free from physical defects and interphase formation. In addition, a charge transfer resistance and a space charge layer resistance at the Li|LPO were not detected. Thus, the changes in the interfacial resistance for different battery SOCs originate from a change in the charge transfer resistance and the space charge layer resistance at the LPO|LCO interface. Using the Frumkin and Langmuir isotherm model,^55^ which considers only the thermodynamics but not the kinetics, the LPO|LCO interfacial charge transfer resistance (*R*_ct_) can be expressed by^56–60^

4 $$R_{ct,LPO|LCO}=\frac{n}{{\left(1-x\right)}^{0.5}x^{0.5}}\exp\left(0.5\times gx\right)$$

Here, *x* corresponds to the Li-content in Li_*x*_CoO_2_, and *n* is a constant under equilibrium state related to the Faraday constant, gas constant, temperature, surface area of the electrode, maximum Li-ion insertion concentration in LCO, bulk Li-ion concentration in LPO, standard Li-ion insertion reaction rate constant, and standard electrode potential under equilibrium. The parameter *g* is related to the interaction energy between two adjacent intercalated Li-ions in LCO (more details are provided in our Supplementary Note 6). Using eq 4, we plot *R*_ct_ vs *x* in Li_*x*_CoO_2_ (Figure 6d). In the range of *x* from 1 to 0.4974, corresponding to the battery voltage up to around 4.3 V vs Li/Li^+^, *R*_ct_ decreases monotonically with increasing *x* in the range of 1 < *x* < 0.5 (inset of Figure 6d). The theoretically calculated LPO|LCO interfacial charge transfer resistance has a minimum at *x* = 0.5. However, our in situ EIS results on the Li|LPO|LCO thin-film battery indicate a minimum in the interfacial resistance at *x* ≈ 0.665 (Figure 6c). The difference in *x* for the respective minimum resistance results from a dynamically changing space charge layer at the LPO|LCO interface. From the difference in the *x* dependency of the interfacial resistance and the theoretical charge transfer resistance, we conclude that the space charge layer resistance must increase monotonically for voltages greater than 3.85 V vs Li/Li^+^ (Supplementary Figure 25, Tables 1, 3, and 4, and Supplementary Note 7). This conclusion is consistent with our space charge layer calculation results in Figure 4f.

For battery voltages of <3.85 V vs Li/Li^+^, there is no significant Li-ion extraction/insertion in bulk LCO. In this case, the interfacial resistance for the LCO|LPO interface is infinite due to the ion-blocking properties of the LCO electrode. Therefore, we have only considered voltages of >3.85 V vs Li/Li^+^ where Li-ions cross the LCO|LPO interface. We calculate the space charge layer resistance by

5 $$R_{scl@U}=R_{\mathrm{int}@U}-R_{ct@U}$$

where at a battery voltage of *U*, *R*_scl@U_ is the LPO|LCO interfacial space charge layer resistance, *R*_ct@U_ is the LPO|LCO interfacial theoretical charge transfer resistance, and *R*_int@U_ is the LPO|LCO total interfacial resistance, respectively.

Based on our finding that the space charge layer resistance monotonically increases within the battery voltage window from 3.85 to 4.3 V vs Li/Li^+^ (details of the calculations are provided in Supplementary Figures 26 and 27, Tables 4–6, and Supplementary Note 8), the results show that the space charge layer resistance continuously increases from around 4.4–5.9 Ω cm^2^ at 3.93 V vs Li/Li^+^ to around 18.4–19.1 Ω cm^2^ at 4.3 V vs Li/Li^+^ (Figure 6e). In addition, we calculated the proportion of the space charge layer resistance in the whole battery interfacial resistance, which increases from 29–39% at 3.93 V vs Li/Li^+^ to around 92–95% at 4.3 V vs Li/Li^+^. The ratio of the space charge layer resistance to the bulk LPO solid electrolyte resistance has a maximum value of only around 6.6% at 4.3 V vs Li/Li^+^ (Figure 6f).

## 3 Conclusions

Our results are applicable to today’s and the next generation ASSBs in two ways: First, we studied a role model thin-film ASSB by using Li|LPO|LCO. We found that a space charge layer mainly plays a role at the interface between the solid electrolyte and the positive electrode. The width of the space charge layer is less than 50 nm. The space charge layer significantly depends on the battery’s SOC. The entire space charge layer evolution relates mainly to a Li-ion redistribution on both sides of the interface. The resistance originating from the space charge layer, which is smaller than 20 Ω cm^2^, accounts for up to 90% of the entire battery interfacial resistance in the operating window of the battery up to 4.3 V vs Li/Li^+^. This space charge layer resistance is expected to further increase with increasing battery voltage, which would play a more important role for high voltage batteries, where the operating window can exceed 5 V vs Li/Li^+^. Possibly, an increasing space charge layer can be compensated by adding a layer that creates an interfacial dipole. This could be a thin ferroelectric interlayer that creates a reverse built-in electric field.^15^ However, the space charge layer resistance accounts for less than 7% of the resistance of the bulk LPO solid electrolyte, which in our case is only 2.5 μm thick. Thus, the resistance of the bulk solid electrolyte dominates over the resistance of the space charge layer in the Li|LPO|LCO thin-film battery. Interphases, with similar ionic conductivity as LPO, usually form between positive electrode materials and most kinds of solid electrolytes.^61–64^ Then a space charge layer forms at the interface between the positive electrode and the newly formed interphase. Thus, in general, a space charge layer resistance in the range of several tens of Ω cm^2^ establishes a reference value for other kinds of solid electrolytes with higher ionic conductivity, such as sulfide. While this study elucidates the behavior of the space charge layer under equilibrium, its evolution under nonequilibrium conditions warrants further investigation. Cycling at different speeds will introduce kinetic limitations that could alter the charge distribution at the interface. Similarly, temperature will directly influence the ionic transport properties of the materials. Furthermore, transitioning from model thin films to particle-based positive electrodes introduces additional complexity. The various exposed crystallographic orientations of particles will present different surface energies and Li-ion diffusion pathways; the size of the particle will change the contact with the solid electrolyte. All of them will affect the formation and evolution of the interfacial space charge layer.

We implemented two operando techniques, heterodyne KPFM and NRA, to study ASSBs. Both the techniques are not commonly used in battery research, although they can reveal valuable interfacial information about the behavior of ASSBs. We were able to show that the combination of both techniques allowed us to locate and quantify the role of space charge layers in ASSBs. In the future, both techniques can potentially be used to investigate a variety of solid electrolyte-based systems, even with additional interphase formation at the interface. With the help of operando heterodyne KPFM and NRA techniques, a quantitative understanding of the interphase and space charge layer evolution could be achieved. Understanding performance-limiting phenomena at interfaces is the key to developing strategies to further improve the performance of ASSBs.

## 4 Methods

### 4.1 Thin-Film Preparation and Electrochemical Characterization

All thin-film depositions and sample transfers were conducted without exposure to air to prevent contamination of surfaces and interfaces. We set up two types of substrates for thin-film deposition. For the operando KPFM, we used a 0.2 mm-thick and 10 mm-square-sized Al_2_O_3_ (0001) single crystalline substrate (Shinkosha Corp.). For the operando NRA measurements, we used a silicon substrate with a chemically etched 1 μm-thick Si_3_N_4_ window (NTT Advanced Technology Corp.). The silicon substrate size and thickness are 10 mm square and 0.625 mm, respectively. First, a Au current collector (200 nm thickness) was plated on a substrate by DC magnetron sputtering (argon pressure of 0.74 Pa and power of 20 W to the 1 in. Au target). Then, a LCO layer (200 nm thickness) as the positive electrode was deposited on the Au layer by pulsed laser deposition (PLD) at a substrate temperature of 600 °C, an oxygen partial pressure of 0.67 Pa, a repetition rate of 10 Hz, and a laser fluence of 1.2 J cm^–2^ with KrF excimer laser using a Li_1.2_CoO_*y*_ target (Toshima Manufacturing Co. Ltd.). Next, a layer of LPO as a solid electrolyte (2.5 μm thickness) is deposited by PLD with an ArF excimer laser (laser fluence of 1 J cm^–2^, vacuum level of <1 × 10^–7^ Torr). Finally, Li metal as a negative electrode (2 μm thickness) was deposited by vacuum thermal evaporation at room temperature. The test cell Li|LPO|Au was prepared in the same way on an Al_2_O_3_ substrate, with the only difference being that a LCO layer was not deposited. We applied a positive voltage to the Au layer to dissolve Li and deposit it on the Au layer. There, Li_*x*_Au alloy forms, and we obtained a cell structure that corresponds to that of Li|LPO|Li_*x*_Au.

All fabricated thin-film batteries were sealed in a vacuum vessel and transferred to an argon-filled glovebox (Mega 3, GS), where sample preparation and electrochemical characterization were conducted (*p*(H_2_O)/*p* < 0.1 ppm and *p*(O_2_)/*p* < 0.1 ppm). All electrochemical characterizations of thin-film batteries, including CV, constant current charging–discharging, and electrochemical impendence spectroscopy (EIS), were performed in the glovebox with a potentiostat (SP150, Biologic) at room temperature.

### 4.2 Argon Ion Milling Polishing

For heterodyne KPFM experiments, we broke the thin-film battery in two halves along the scratch, which we made in the backside of the Al_2_O_3_ substrate. One half of the battery was fixed in a cross-section milling holder. Next, the sealed milling holder was transferred to the argon ion milling chamber (IM4000, Hitachi). Then, we polished the cross-section of the thin-film battery by argon ion milling in pulsed mode. An acceleration voltage of 2 V was applied to generate an argon ion beam for 40 s each for 2 min. The corresponding ion current was around 450 μA. While polishing, the rotating stage was set to a speed of 30 rpm/min. The whole polishing process took around 24 h. After argon ion milling, the sealed milling holder and the polished battery were both transferred back to the glovebox. All further sample handling and preparation steps were done in the argon gas-filled glovebox (Mega 3, GS).

### 4.3 KPFM Measurements

Inside the glovebox, we placed the polished battery in a homemade poly­(ether-ether-ketone) (PEEK) holder, which allowed us to connect the battery to a potentiostat (SP150, Biologic) while performing KPFM on the polished cross-section of the thin-film battery. Heterodyne KPFM was performed with a scanning force microscope (MFP-3D Asylum Research, Oxford Instruments) connected to an additional lock-in amplifier (HF2LI 50 MHz, Zurich Instruments). We used heterodyne frequency modulated KPFM to acquire a high lateral and potential resolution.^65^ Heterodyne KPFM can effectively mitigate the influence of the sample’s volumetric fluctuations and surface roughness by the usage of a phase-locked loop. The electrostatic forces are demodulated at a sideband frequency far away from the resonance peak. This approach results in less AC crosstalk compared with other KPFM techniques.^65^ For KPFM, we used Pt/Ir-coated cantilevers, which have a nominal spring constant of 2 N/m and a nominal resonance frequency of 75 kHz (SCM-PIT-V2, Bruker). All heterodyne KPFM measurements were performed in an argon-filled glovebox to prevent degradation of the metal Li negative electrode and contamination of the polished thin-film battery cross-section. During KPFM measurement, the mechanical excitation frequency was 63 kHz, the modulation frequency was 345 kHz, the applied AC voltage was 2 V, and the scan rate was 0.1 Hz. For operando heterodyne KPFM testing, we connected a potentiostat to the thin-film battery to charge and discharge the battery to different states of charge (SOC). The Li negative electrode acts as a counter electrode and was grounded. Au was used as a current collector and was connected to the working electrode.

### 4.4 SEM and EDS Measurements

A field-emission SEM instrument (U900, Hitachi) was used to image the morphology of the polished cross-section of a thin-film battery. An EDS (Oxford Instruments) enabled the characterization of the surface element distribution on the polished cross-section. To avoid contamination of the polished surface and chemical reactions after exposure to air, an air protection transfer box filled with argon gas was used to transfer the battery from the glovebox into the SEM chamber.

### 4.5 NRA Measurements

NRA and Rutherford backscattering (RBS) techniques were used for operando compositional depth profiling. The depth resolution near the LPO-LCO interface was ∼50 nm. NRA is based on a H^+^ ion beam, which is irradiated from the substrate side into the thin-film battery. Then H^+^ ions react with Li and generate α-particle radiation. The counts and the energy of α-particles reflect the relative Li density at a specific sample depth. Thin-film batteries were fixed in a sealed electrochemical cell in the glovebox and evacuated to a pressure <5 × 10^–4^ Pa. Then the electrochemical cell was transferred to the vacuum chamber of the ion-beam irradiation line (vacuum level of <1 × 10^–4^ Pa at the 1 MV Tandem Accelerator, Univ. Tsukuba, Japan). We used 1 MeV H^+^ beams for the NRA and RBS measurements.

The raw NRA spectra (counts vs energy) were converted to a depth spectrum (counts vs depth) based on calculated depth profiles of the conversion factor with the nuclear reaction cross-section of each element. The nuclear reaction cross-section of Li is calculated from the experimental NRA and RBS spectra of a LiNbO_3_ (0001) single crystal. We simulated the depth spectrum with Gaussian distributions, allowing for multiple peaks corresponding to a multilayer model. We considered the decreasing resolution with beam penetration depth by assigning 1, 16, and 3 Gaussian distributions for Li, LPO, and LCO layers, respectively.

### 4.6 Synchrotron X-ray Photoemission Spectroscopy

The electronic band structures of the LPO solid electrolyte and the LCO positive electrode were determined by using synchrotron X-ray photoemission spectroscopy. Thin films were sealed in a vacuum box and directly transferred to the synchrotron undulator beamline (BL-2A, Photon Factory, KEK, Tsukuba). The Fermi level was aligned by using the Au 4f peak.

### 4.7 Space Charge Layer Theoretical Calculation

The Li-ion concentration profile in the LPO|LCO interfacial space charge layer was calculated based on Niek J. J. de Klerk’s space charge layer model.^53^ In our calculation, we assumed that (1) the evolution of a space charge layer at an interface must obey mass conservation, (2) Li-ion is the only kind of charge that can migrate across LPO|LCO interfaces, and (3) all the calculations are based on an equilibrium state over 3.85 V vs Li/Li^+^ when Li-ions can be extracted from LCO (All space charge model parameters are listed in Supplementary Table 2).

### 4.8 LCO Work Function Calculation

Computations were performed using density function theory (DFT) with the Perdew–Burke–Ernzerhof (PBE) functional,^66^ as implemented in the Vienna Ab initio Simulation Package (VASP).^67^ The valence electron wave functions were expanded in plane-wave basis sets with a 400 eV cutoff, and the projector augmented wave method was used to describe the core–electron interactions.^68^ A 14 × 5 × 1 *k*-point grid was sampled by using the Monkhorst–Pack scheme for each computation. The effect of the dipole moment on the potential was corrected using the LDIPOL keyword in VASP.

The (100) surface of LiCoO_2_ was created from the supercell (with Li_30_Co_30_O_60_ as the unit cell) of the experimental crystal structure with a 15 Å vacuum slab.^69^ To create the Li_*x*_CoO_2_ structures, we gradually removed Li from the interior layer (bulk environment). All such created structures were fully optimized until the residual forces on the constituent atoms became less than 0.02 eV Å^–1^, while the lattice parameters were kept fixed.

To calculate the work function, the electrostatic potentials *V*(*x*,*y*,*z*) on a dense grid were first computed using VASP. Then *V*(*x*,*y*,*z*) of the same *z* positions were averaged (i.e., averaged over *xy* planes), leading to *E*(*z*). The work function can then be calculated using

6 $$WF=E-E_{F}$$

where *E* is the averaged electrostatic potential energy in a vacuum and *E*_F_ is the Fermi energy of Li_*x*_CoO_2_.

## Supplementary Material



## Notes

Details of the thin-film structure, SXPS spectra, CV measurements, cycling performance, supporting KPFM measurements, DFT calculation details, details of the energy of electronic bands for different materials, additional supporting NRA spectra, RBS, space charge layer calculations at the LPO|LCO interface, EIS measurements and fitting results, a diagram with interfacial resistance and theoretical interfacial charge transfer resistance as a function of battery voltage, and tables with material parameters used for calculations and a detailed description of Heterodyne KPFM measurements (PDF)
