# Supplementary material for: Space Charge Layer Evolution in All-Solid-State Batteries Probed via Operando Kelvin Probe Force Microscopy and Nuclear Reaction Analysis
Source: ACS Nano. 2025 Nov 5;19(45):39062–75. doi: 10.1021/acsnano.5c10125 (PMC12632181; doi:10.1021/acsnano.5c10125)
Supplement: Supplementary file 1 [file nn5c10125_si_001.pdf]

Supplementary Information

Space Charge Layer Evolution in All-Solid-State Batteries Probed via Operando Kelvin Probe Force Microscopy and Nuclear Reaction Analysis

Authors:

Chao Zhu[1+], Shigeru Kobayashi[2+], Yuki Sugisawa[3], Franjo Weber[1], Kun-Han Lin[1,7], Miho Kitamura[4], Koji Horiba[4], Hiroshi Kumigashira[5], Kazunori Nishio[6], Ryota Shimizu[2], Daiichiro Sekiba[3], Taro Hitosugi[2*], Rüdiger Berger[1*]

Addresses:

1. *Max Planck Institute for Polymer Research, Ackermannweg 10, 55128 Mainz, Germany*
2. *Department of Chemistry, The University of Tokyo, Tokyo 113-0033, Japan*
3. *Graduate School of Pure and Applied Sciences, University of Tsukuba, Tsukuba, Ibaraki 305-8573, Japan*
4. *National Institutes for Quantum Science and Technology (QST), Sendai 980-8579, Japan*
5. *Institute of Multidisciplinary Research for Advanced Materials (IMRAM), Tohoku University, Sendai, Miyagi 980-8577, Japan*
6. *School of Materials and Chemical Technology, Tokyo Institute of Technology, Tokyo 152-8552, Japan*
7. *Department of Chemical Engineering, National Tsing Hua University, Hsinchu 300044, Taiwan*

+ These two authors contributed equally

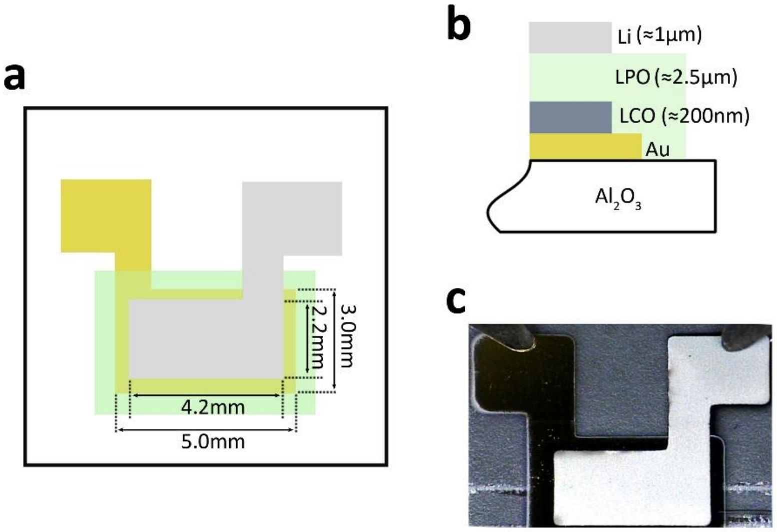

Supplementary Figure 1. Structure of a thin-film battery. (a) Morphology and size of our Li | LPO | LCO |Au thin-film model battery. (b) Schematic diagram of the thickness of different layers in our thin-film battery. (c) Photo of the prepared thin-film battery.

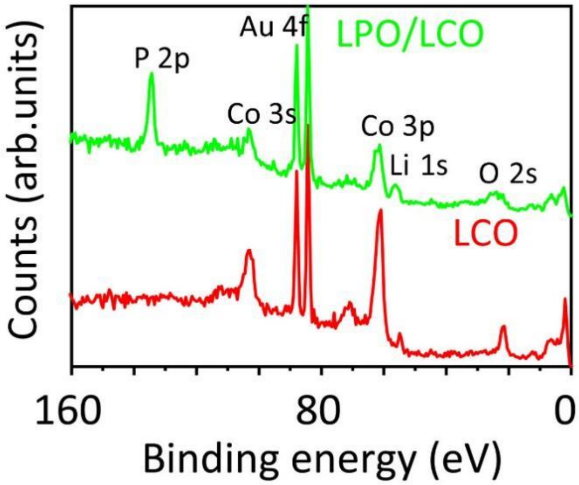

Supplementary Figure 2. SXPS spectra (binding energy from 0 to 160 eV). SXPS comparison of pure LCO and LCO coated with a thin LPO film.

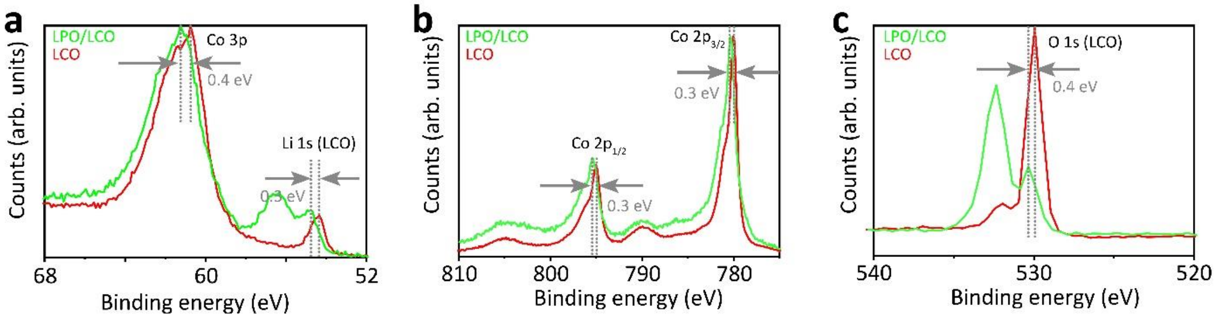

Supplementary Figure 3. More detailed SXPS spectra. Comparison of pure LCO and LCO coated with LPO at the binding energies of (a) Co 3p, Li 1s (LCO), (b) Co $2p_{1/2}$, Co $2p_{3/2}$ and (C) O 1s (LCO).

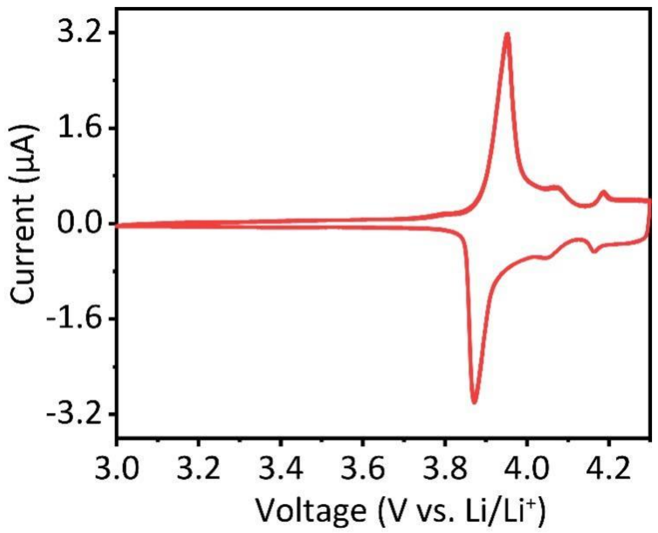

Supplementary Figure 4. CV measurements of a thin-film battery at 1 mV s^{-1}.

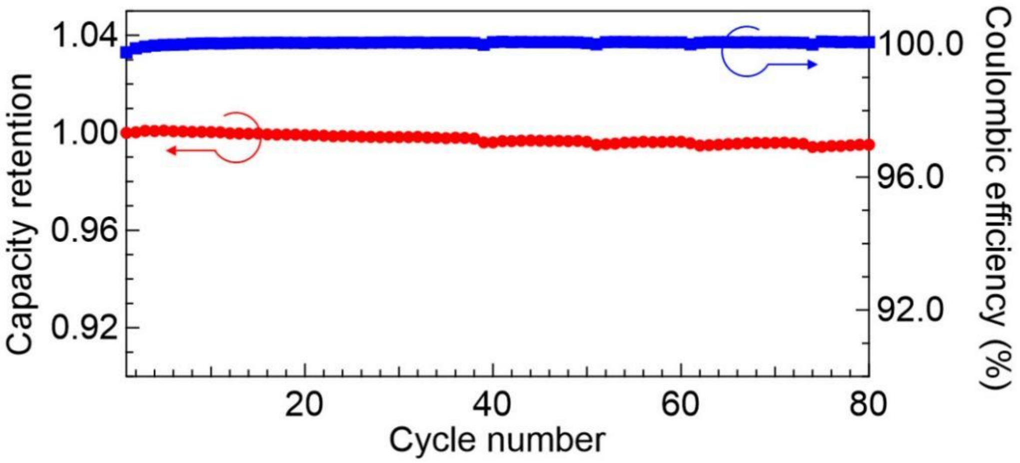

Supplementary Figure 5. Cycling performance of a thin-film battery.

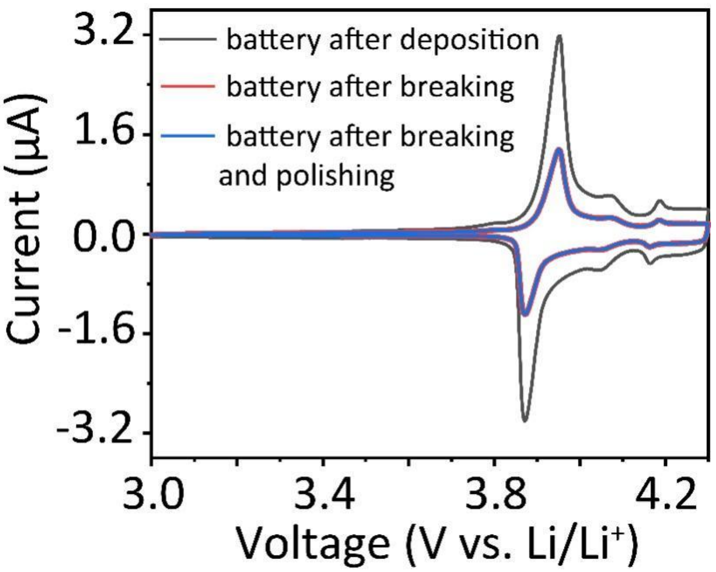

Supplementary Figure 6. Comparison of CV curves. CV curves of the same thin-film battery after deposition and before breaking (black line), after breaking in two halves (red line), and after polishing the broken battery (blue line). The CV curves were recorded at 1 mV/s scan speed. After breaking the batteries, the current was halved owing to a 50 % reduction in battery area. The CV curves of the battery after breaking (red line) and after polishing (blue line) overlap.

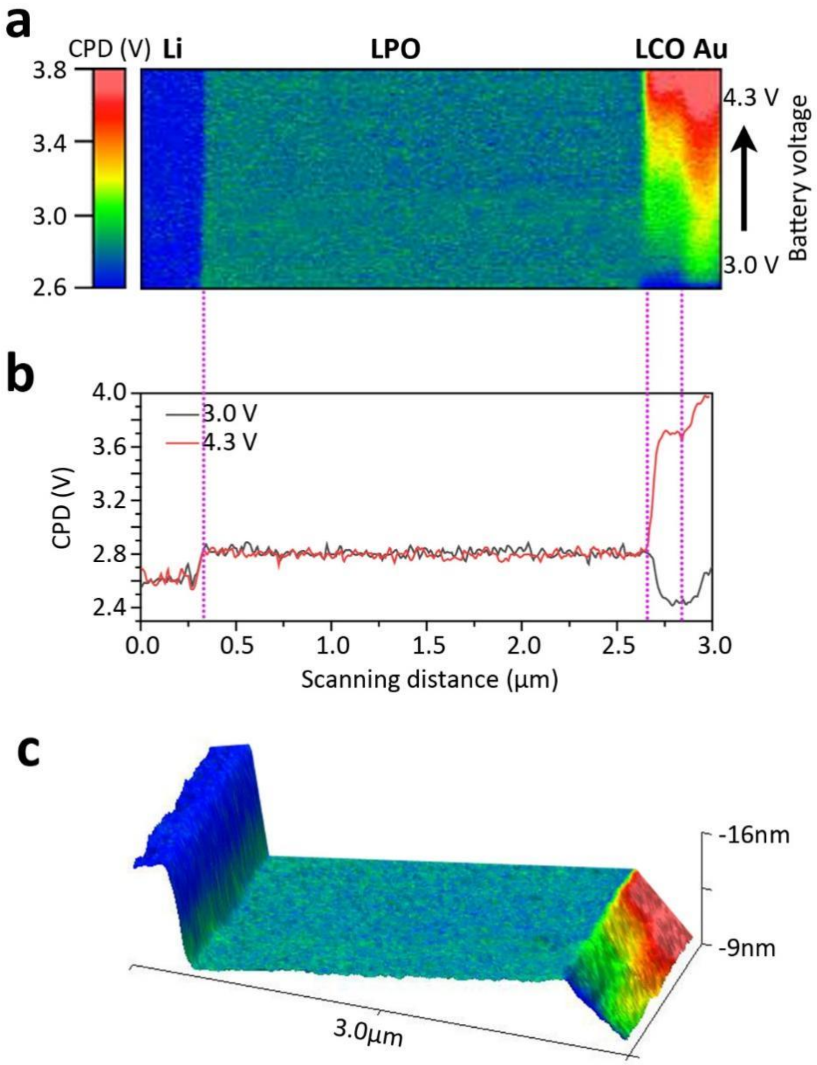

Supplementary Figure 7. Heterodyne KPFM measurements of the whole battery cross-section taken while the battery was being charged. (a) Evolution of the CPD including Li layer (left side), LPO layer (middle), LCO layer and Au layer (right side) while increasing the battery voltage from 3.0 V to 4.3 V vs. Li/Li^{+}. (b) CPD line profiles extracted from the CPD map in (a) at a battery voltage of 3.0 V vs. Li/Li^{+} (black line) and at 4.3 V vs. Li/Li^{+} (red line), respectively. (c) 3-dimensional representation of the topography of the area measured in (a). We added a skin layer on the 3-dimensional topography view to represent the local CPD.

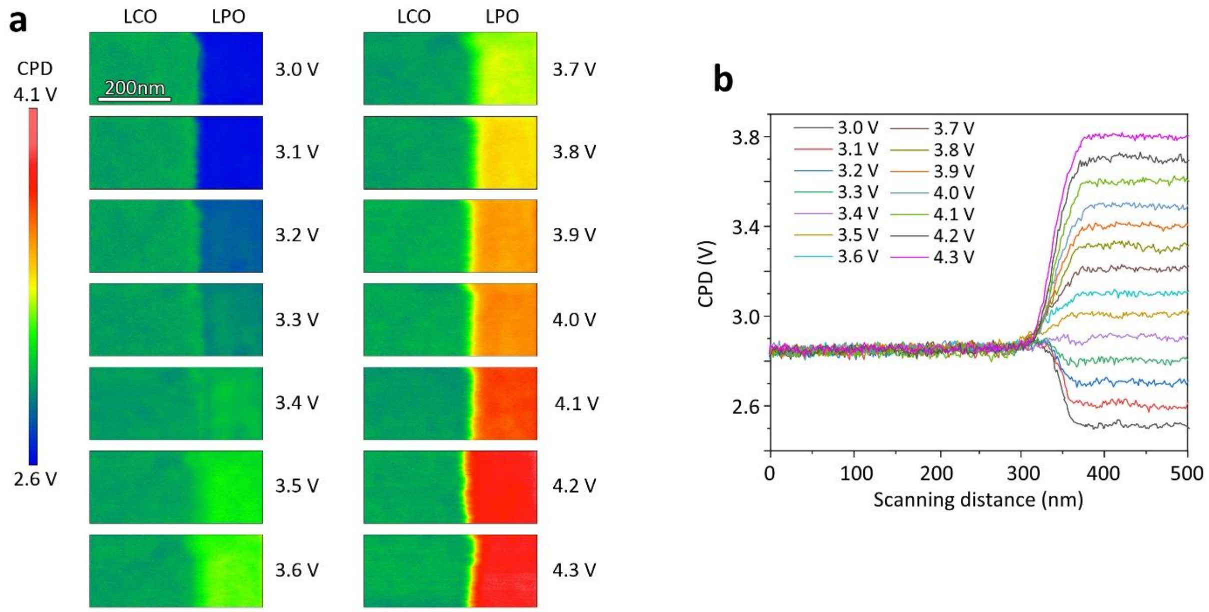

Supplementary Figure 8. CPD map at the LPO|LCO interface at different voltages. (a) Close-up of the CPD evolution of the LPO|LCO interface and adjacent areas at changing battery voltages after the battery was cycled 80 times. (b) CPD line profiles extracted from CPD maps in (a) under different battery voltages.

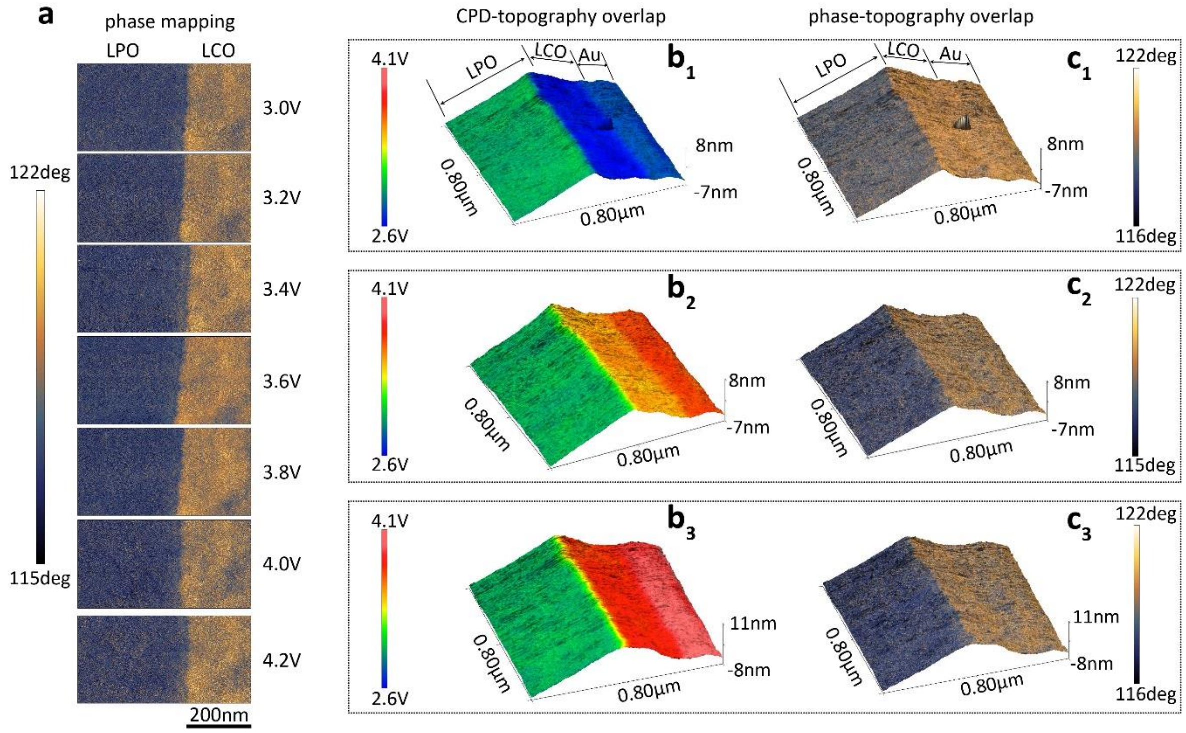

Supplementary Figure 9. Topography effect on the CPD map at the LPO|LCO interface. (a) Scanning force microscopy phase changes of the resonating cantilever at the LCO|LPO interface at different battery voltages. Different battery voltages do not change the phase of the resonating cantilever. To visualize where the LCO|LPO interface is located, we plotted the topography as a 3-dimensional illustration and overlayed the corresponding CPD signals (b_1 – b_3) and phase changes (c_1 - c_3) as colored skin layers. The three dotted boxes indicate different battery voltages of 3.0 V vs. Li/Li^+, 3.8 V vs. Li/Li^+ and 4.3 V vs. Li/Li^+, respectively. All illustrations prove that the transition regions in the CPD map and the phase map overlapped with the topography kink at the LPO|LCO interface.

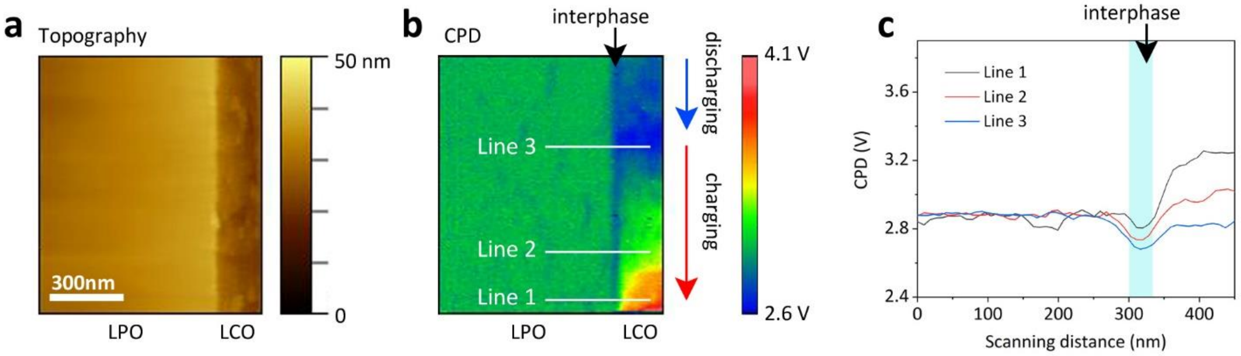

Supplementary Figure 10. Heterodyne KPFM measurements on a thin-film battery with interphase formation at the LPO|LCO interface caused by poorly controlled vacuum atmosphere conditions during preparation. (a) Topography and (b) CPD map of LPO and LCO at different charging and discharging states. (c)Line profiles extracted from white line 1-3 from the CPD map in (b).

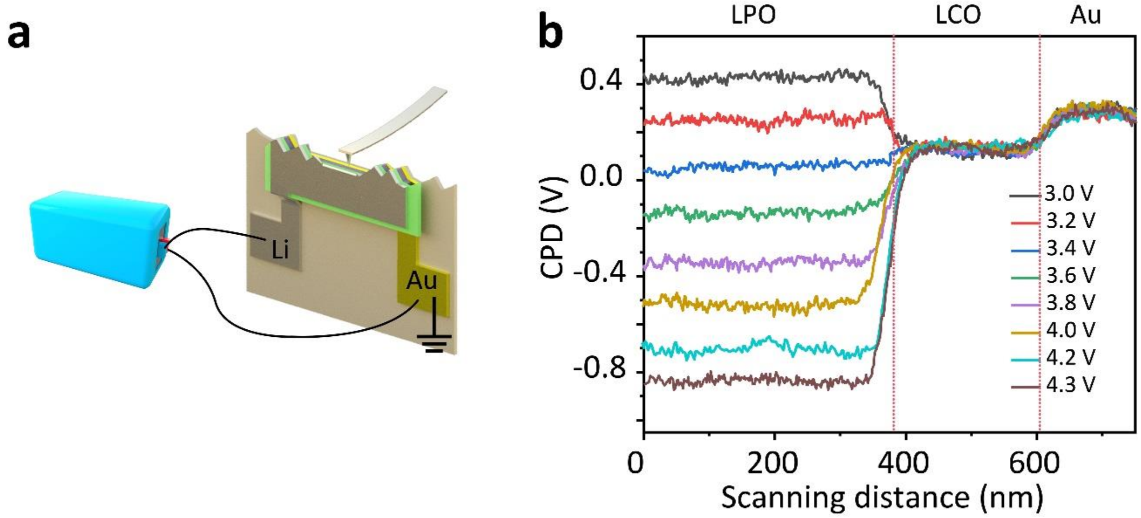

Supplementary Figure 11. **Heterodyne KPFM measurements on a thin-film Au grounded electrode.** (a) Schematic drawing of the KPFM measurement on the cross-section of a thin-film battery. The Li electrode is connected to the working electrode and the Au layer is connected to the counter electrode of the potentiostat and grounded. (b) CPD line profiles recorded along the cross-section of the thin-film battery with the electrical connection shown in (a). We measured different battery voltages ranging from 3.0 to 4.3 V vs. Li/Li^+.

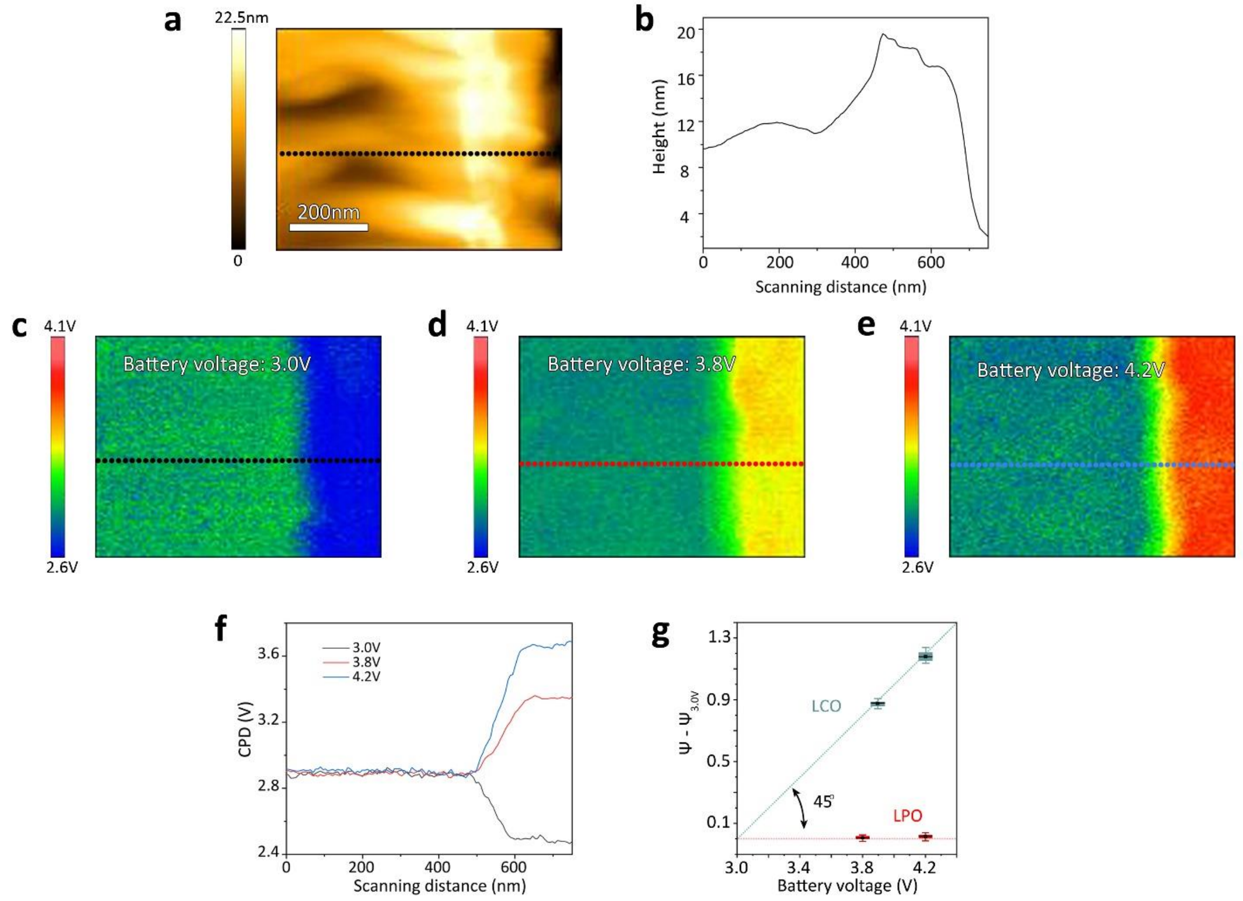

Supplementary Figure 12. Heterodyne KPFM measurements on a thin-film Au grounded electrode on an unpolished thin-film battery cross-section. (a) Topography of a thin-film battery cross-section, which was prepared by breaking only. The surface is much rougher compared to an ion-polished surface. The KPFM-imaged surface includes the LPO and the LCO layers. (b) Topography line profile along dotted line in (a). (c-e) Measured CPD maps of the same surface in (a) at a battery voltage of 3.0, 3.8 and 4.2 V vs. Li/Li^+, respectively. (f) CPD line profiles along dashed lines in (c-e). (g) Volta potential (ψ) mean values change calculated from all the pixels on the LCO and LPO layer surfaces at different battery voltages compared to 3.0 V. In this analysis we excluded the LPO|LCO transition region.

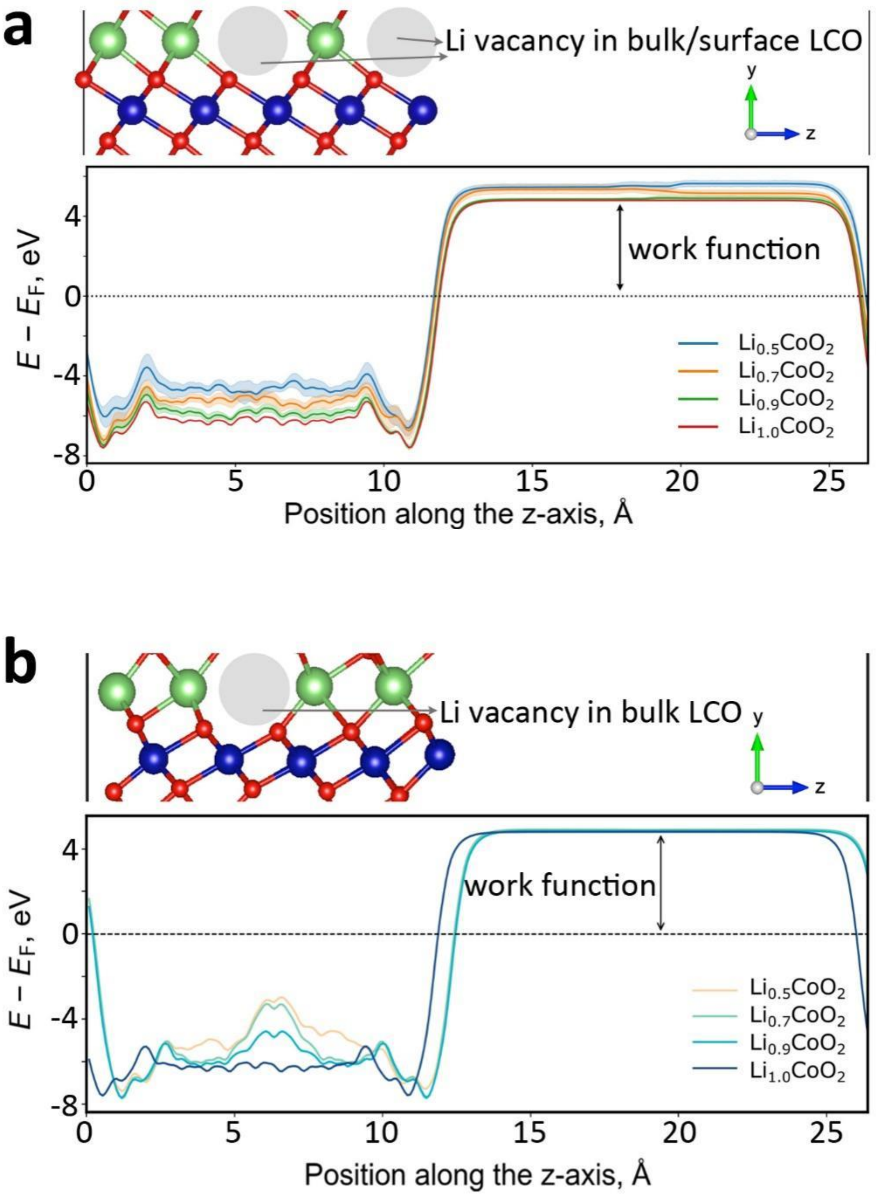

Supplementary Figure 13. DFT calculations of work function on different Li concentrations in LCO. (a) (Top) Illustration of the (100) surface of Li_xCoO_2 with a 15 Å thick vacuum slab. In the simulations, Li atoms are removed from both surface and bulk $LiCoO_2$. (Bottom) The averaged potential energy (subtracted by the Fermi energy) of Li_xCoO_2 as a function of the z position. The work function is obtained from the difference between the vacuum potential energy and the Fermi energy (see computational details). (b) (Top) Illustration of the (100) surface of Li_xCoO_2 with a 15 Å thick vacuum slab, where Li atoms are removed from the bulk but not from the surface of $LiCoO_2$. An example for a Li vacancy, which is left behind (grey spot). (Bottom) The averaged potential energy (subtracted by the Fermi energy) of Li_xCoO_2 as a function of the z position.

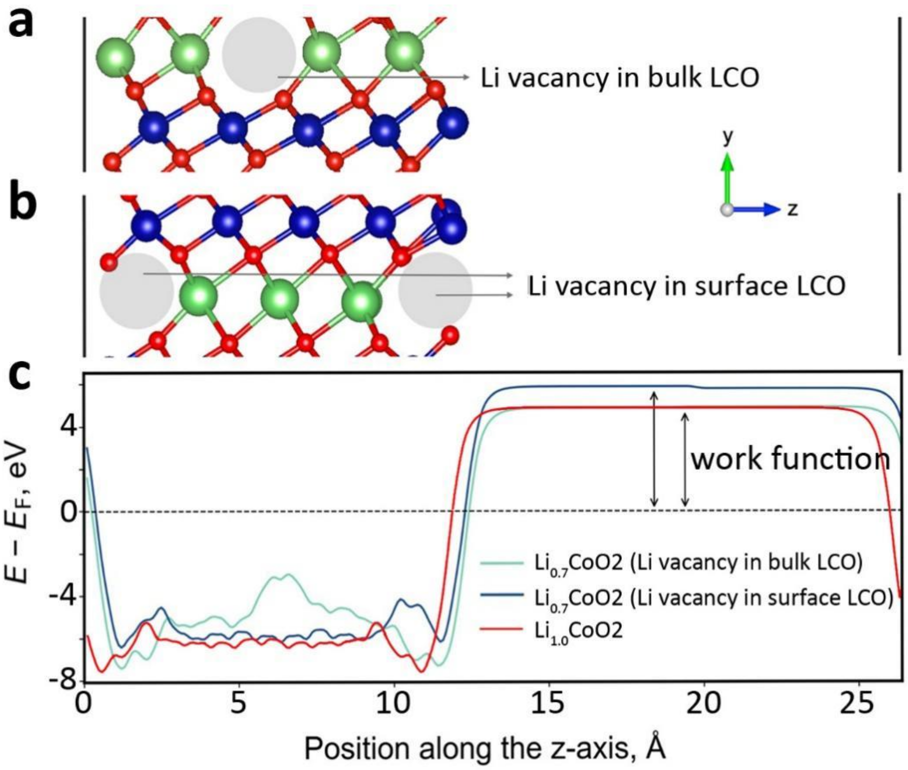

Supplementary Figure 14. DFT calculations comparison of work function on Li-ions extraction from surface and bulk LCO. (a) The illustration of the (100) surface of Li_xCoO_2 with a 15 Å vacuum slab, where Li atoms are removed only from the bulk of $LiCoO_2$. (b) The illustration of the (100) surface of Li_xCoO_2 with a 15 Å vacuum slab, where Li atoms are removed only from the surface of $LiCoO_2$. (c) The average potential energy (subtracted by the Fermi energy) of $LiCoO_2$ and $Li_{0.7}CoO_2$ as a function of the z position. For $Li_{0.7}CoO_2$, there are two states, Li removed from the bulk and from the surface of $LiCoO_2$, are both shown.

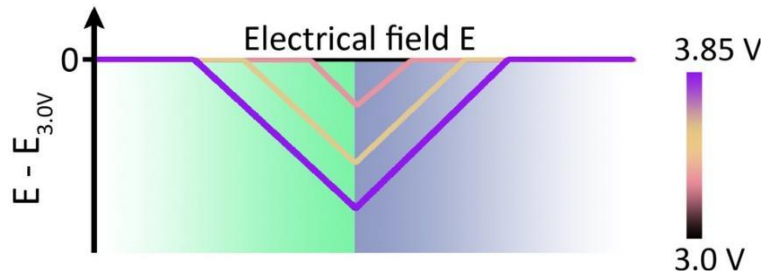

Supplementary Figure 15. Electrical field evolution at the LPO | LCO interface. Schematic of electrical field change at the LPO | LCO interface. All changes are drawn relative to a reference voltage of 3.0 V vs. Li/Li^+ and are valid for a battery voltage range of 3.0 V – 3.85 V vs. Li/Li^+.

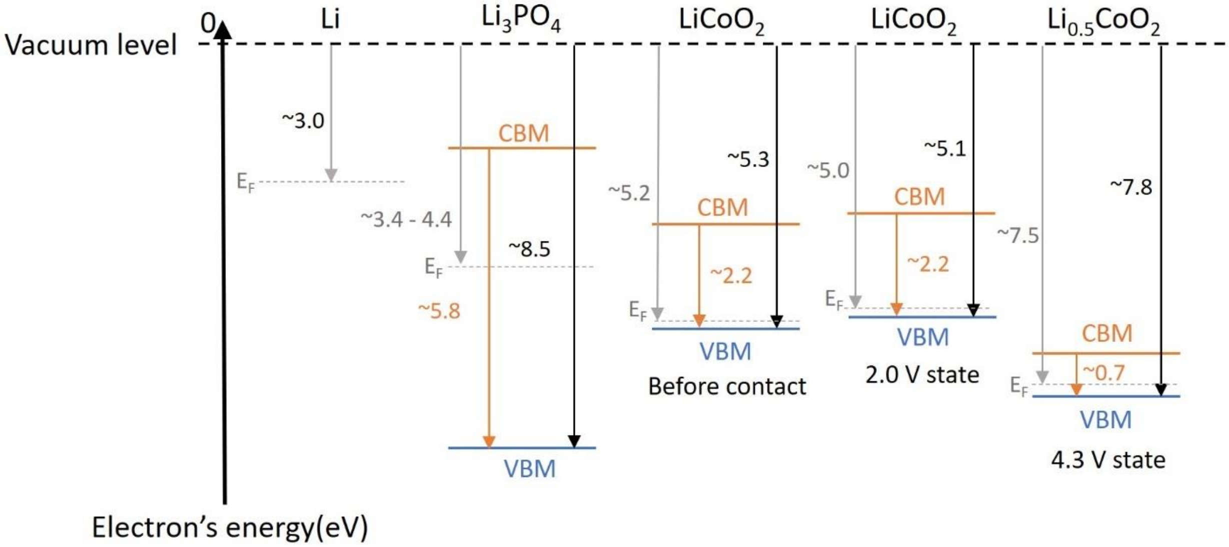

Supplementary Figure 16. Energy of electronic bands for different materials. The energies relative to the vacuum level of the electronic bands of metal Li, Li_3PO_4, $LiCoO_2$ (before contact with Li_3PO_4), and $LiCoO_2$ (2 V vs. Li/Li^+) and $Li_{0.5}CoO_2$ (around 4.3 V vs. Li/Li^+) in a fabricated thin-film battery. Blue lines correspond to valence band maximum (VBM) energies, brown lines correspond to conduction band minimum (CBM) energies and the grey dotted lines correspond to Fermi level (E_f).

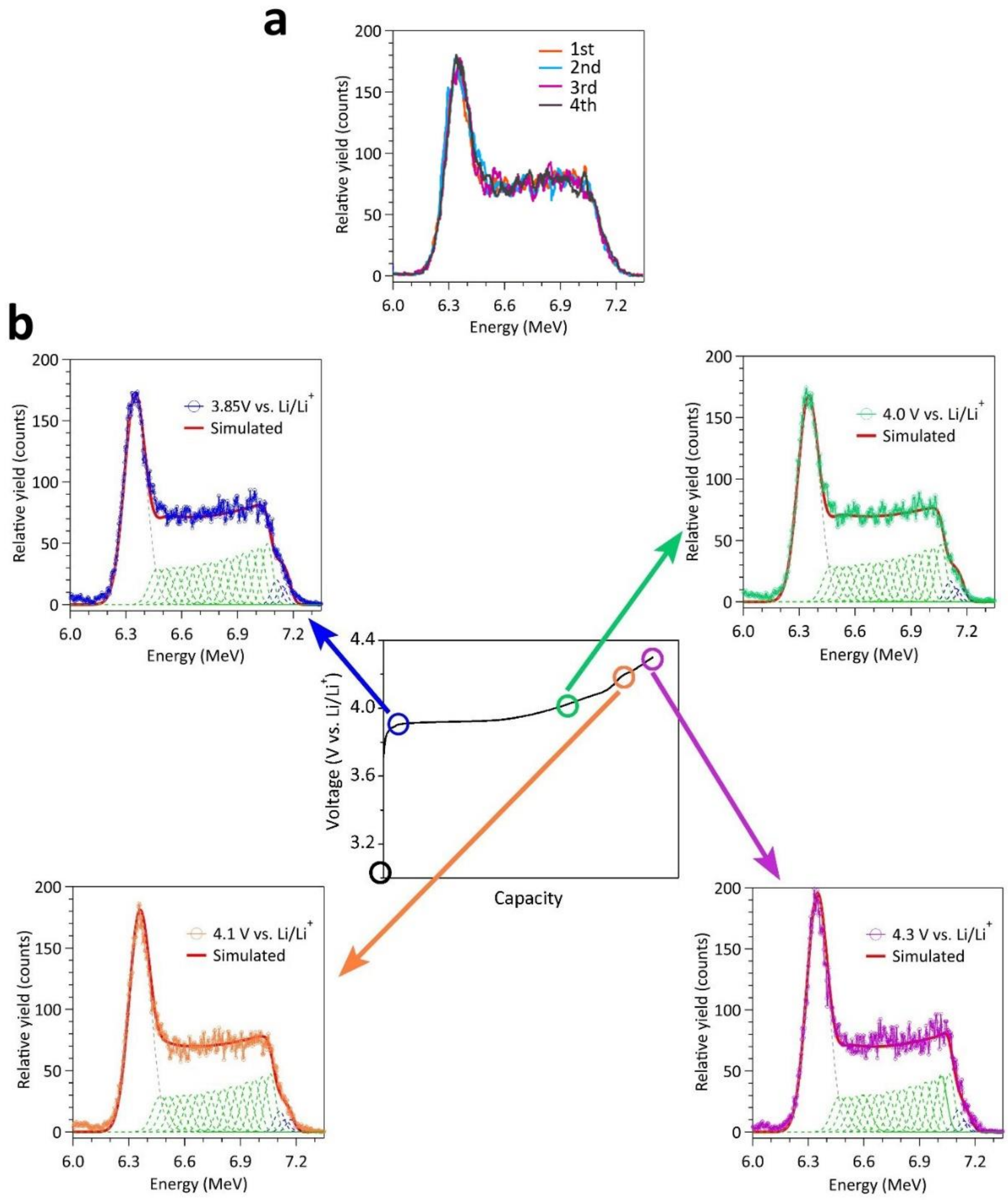

Supplementary Figure 17. (a) Comparison of four separate NRA spectra for the same battery acquired at a constant 3.0 V. (b) NRA spectra for battery voltages of 3.85 V, 4.0 V, 4.1 V and 4.3 V vs. Li/Li^+, respectively.

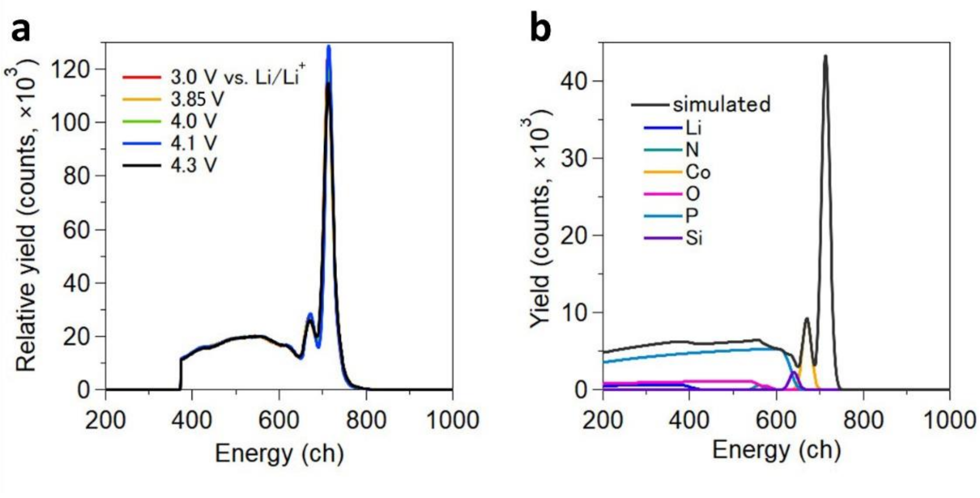

Supplementary Figure 18. RBS results across the whole battery for different battery voltages. (a) Rutherford backscattering spectrometry (RBS) results during the nuclear reaction analysis measurements. (b) Simulated RBS spectra for a thin-film multilayer model. We note that the rough interface or Au-Li alloy layer between the $LiCoO_2$ and Au is required to reproduce the experimental peak structure. The simulation was performed using SIMNRA software.

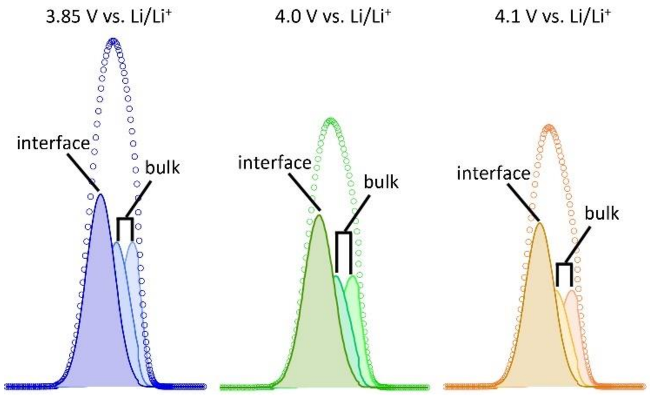

Supplementary Figure 19. Fitting results of NRA curve in the LCO layer. Fit of 3 Gaussian peaks to the signal attributed to the $LiCoO_2$ layer. As examples, we show the results for battery voltages of 3.85 V, 4.0 V and 4.1 V vs. Li/Li^+.

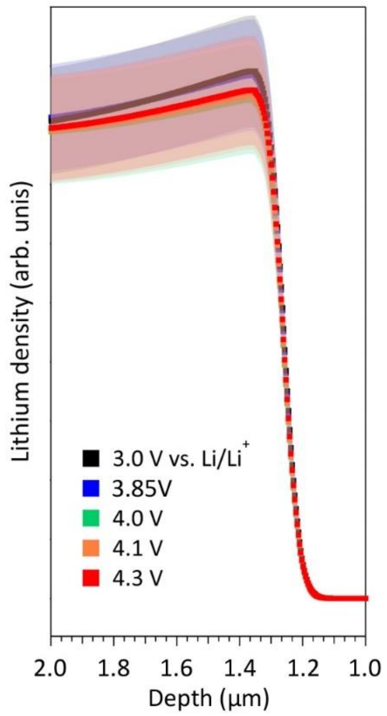

Supplementary Figure 20. The Li content obtained from the fits at the LPO layer for different battery voltages.

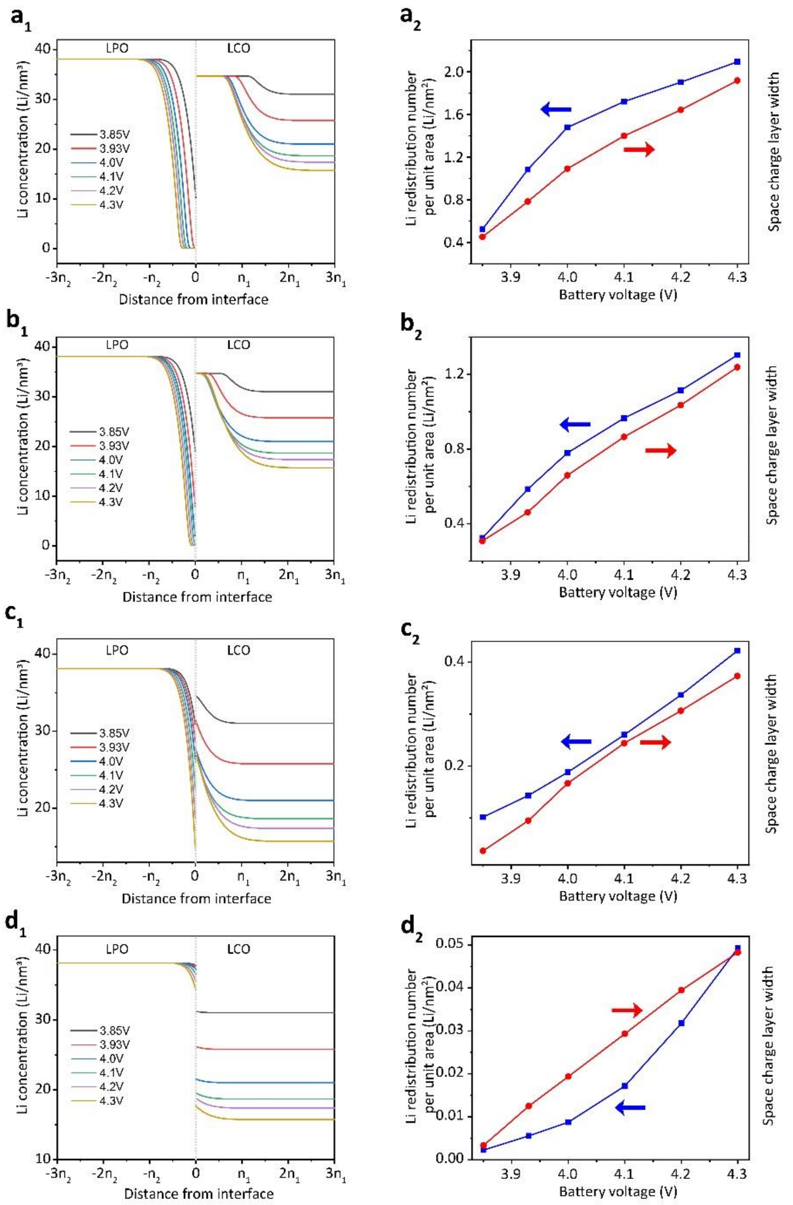

Supplementary Figure 21. Space charge layer calculations at the LPO|LCO interface. Theoretical calculation of the Li-ion concentration distribution at the LPO | LCO interface for different battery voltages vs Li/Li^+ given that the equilibrium potential of LPO vs. Li/Li^+ is (a_1) 1.4 V, (b_1) 2.1 V, (c_1) 2.8 V and (d_1) 3.4 V, respectively. (a_2-d_2) show the corresponding evolution of the number of Li-ions from LPO to LCO for space charge layer formation and width of the space charge layer at the LPO|LCO interface for different battery voltages.

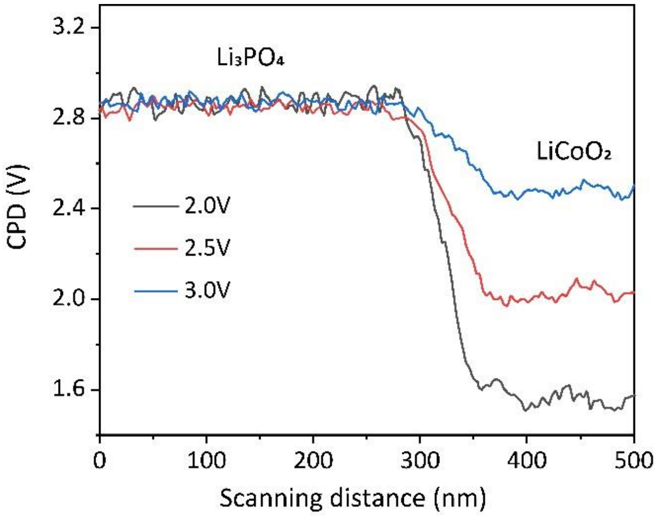

Supplementary Figure 22. CPD line profiles across the LPO|LCO interface at battery voltages of 2.0 V, 2.5 V and 3.0 V vs. Li/Li^+, respectively.

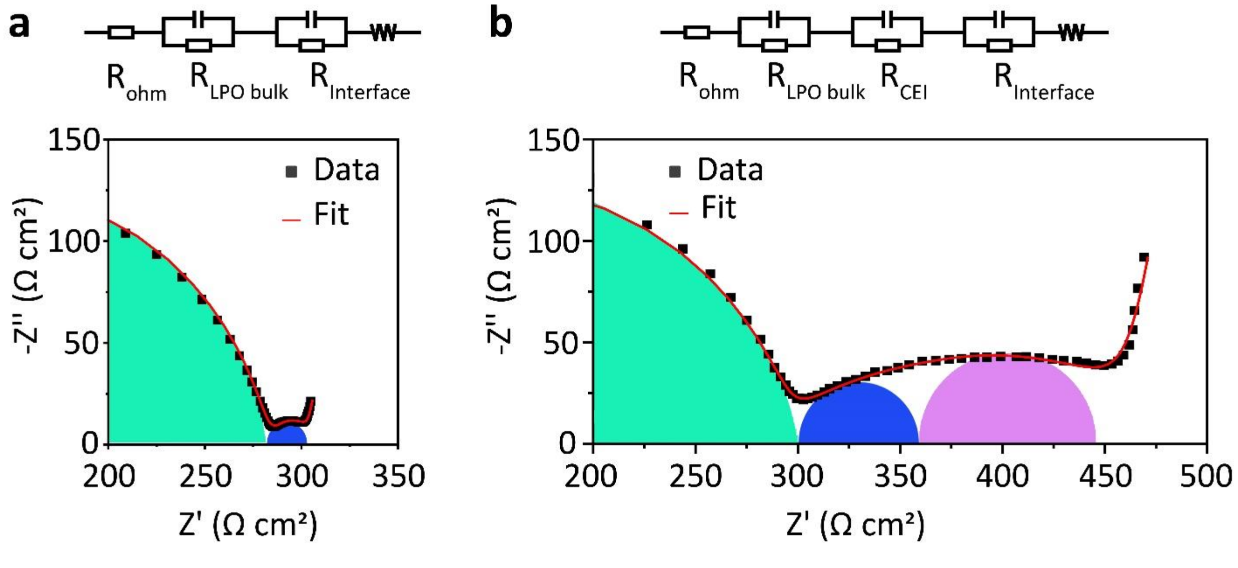

Supplementary Figure 23. Comparison of EIS measurements and fitting results at 4.3 V vs. Li/Li^+ for two thin-film batteries. (a) thin-film battery prepared under a well-controlled vacuum atmosphere, and (b) thin-film battery with the LPO layer deposited with higher fluence.

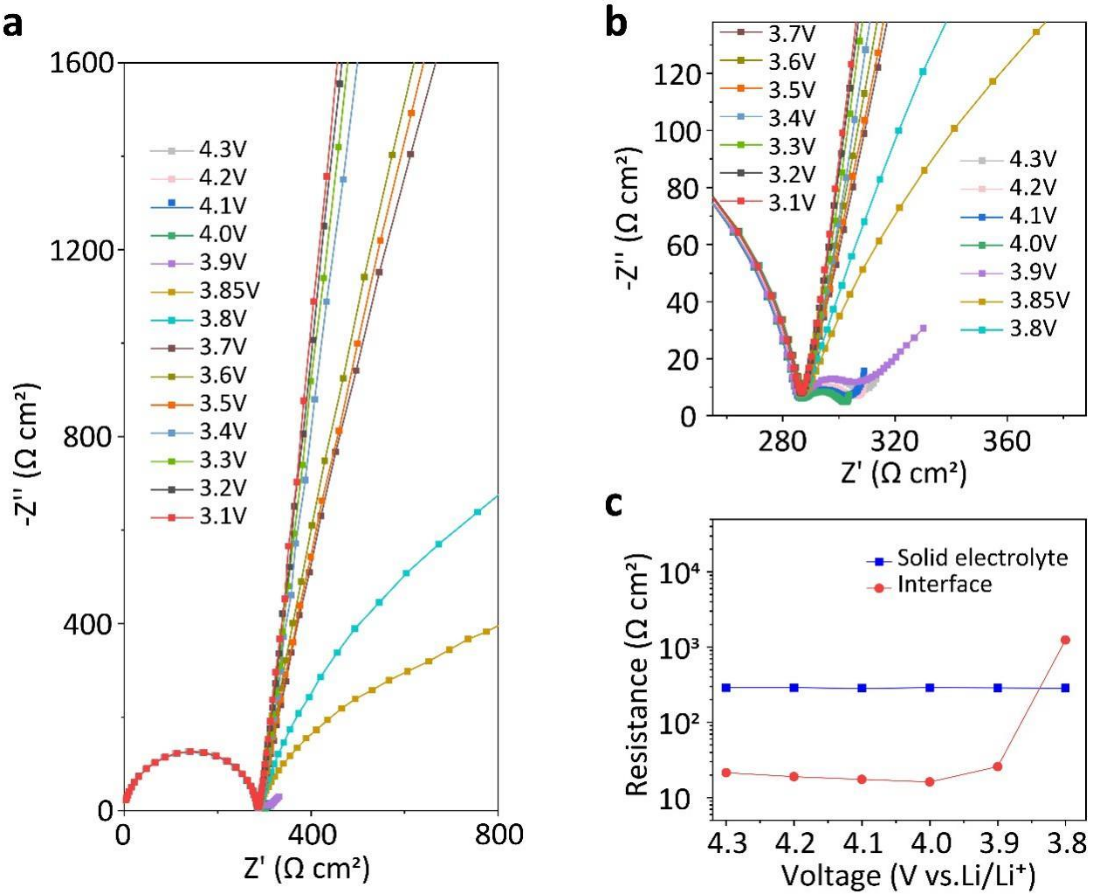

Supplementary Figure 24. EIS of a thin-film battery being discharged from 4.3 V to 3.1 V vs. Li/Li⁺. (a) Nyquist plots of a Li|LPO|LCO thin-film battery at different voltages. (b) Magnification of the Nyquist plots in the high- and mid-frequency range in (a). (c) LPO bulk resistance and interfacial resistances extracted by fitting the data from (b).

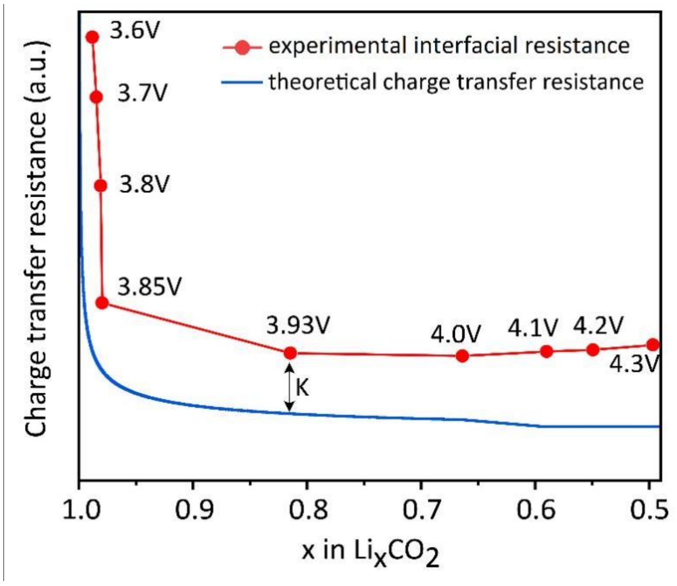

Supplementary Figure 25. LPO|LCO experimental interfacial resistance and theoretical interfacial charge transfer resistance as a function of battery voltage.

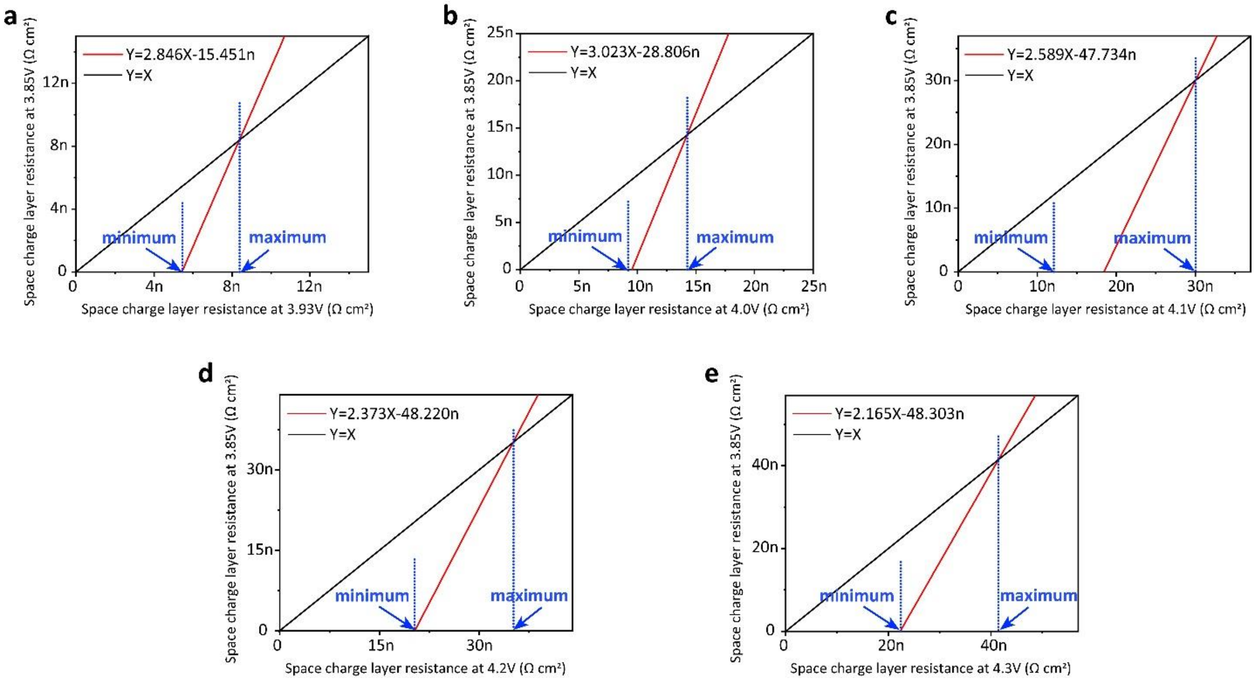

Supplementary Figure 26. Cartesian plots of function Y(X) (equation S24) and Y=X at different battery voltages of: (a) 3.93 V vs. Li/Li^+, (b) 4.0 V vs. Li/Li^+, (c) 4.1 V vs. Li/Li^+, (d) 4.2 V vs. Li/Li^+ and (e) 4.3 V vs. Li/Li^+.

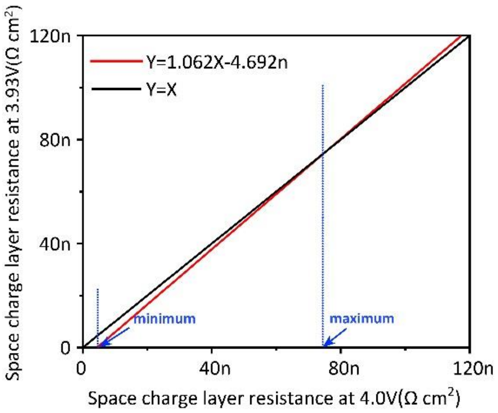

Supplementary Figure 27. Cartesian plots of function $Y_2(X)$ (equation S25) and Y=X at battery voltage of 4.0 V vs. Li/Li^+.

Supplementary Table 1. Li concentration x in Li_xCoO_2 as a function of the battery voltage change

Voltage (V vs. Li/Li$^+$)	3.6	3.7	3.8	3.85	3.92	4.0	4.1	4.2	4.3
**x in Li_xCoO_2**	99.00%	98.65%	98.26%	98.16%	81.59%	66.47%	59.07%	55.00%	49.74%

Supplementary Table 2. Material parameters for the LPO | LCO interfacial space charge layer calculation

Property	LPO	LCO[1]
**C_{max} (Li/nm^3)**	76.28	34.76
**C_0 (Li/nm^3)**	38.14	Depends on voltage
**Voltage (vs. Li/Li$^+$)**	0.7, 1.4, 2.1, 2.8, 3.5	3.83, 3.93, 4.0, 4.1, 4.2, 4.3
**ε (relative to ε_0)**	5.6	12.9
**Ω (eV)**	0[1]	0.026
**E_M (eV/Li)**	5.27[2]	7.00

Supplementary Table 3. LPO | LCO interfacial charge transfer resistance (R_{ct}) and thin-film battery voltages

Voltage (V vs. Li/Li$^+$)	3.6	3.7	3.8	3.85	3.93	4.0	4.1	4.2	4.3
**R_{ct} (Ω)**	72.79n	62.32n	54.58n	52.99n	13.19n	8.00n	2.03n	2.01n	2.00n

Supplementary Table 4. The summary of total interfacial resistance at the LPO | LCO interface in our thin-film battery at different battery voltages.

Voltage (V vs. Li/Li$^+$)	3.85	3.93	4.0	4.1	4.2	4.3
**R_{int} (Ω cm^2)**	43.34	15.23	14.34	16.74	18.26	20.02

Supplementary Table 5. The summary of maximum R_{scl} and minimum R_{scl} at the LPO | LCO interface in our thin-film battery at different battery voltages.

**Voltage (V vs. Li/Li$^+$)**	3.93	4.0	4.1	4.2	4.3
$\boldsymbol{R_{scl,max}}$ **(Ω)**	8.37n	14.24n	30.04n	35.12n	41.46n
$\boldsymbol{R_{scl,min}}$ **(Ω)**	5.43n	9.53n	18.44n	20.32n	22.31n

Supplementary Table 6. Summary of the exact maximum and minimum R_{scl} for different voltages at the LPO | LCO interface

**Voltage (V vs. Li/Li$^+$)**	3.93	4.0	4.1	4.2	4.3
$\boldsymbol{R_{scl,max}}$ **(Ω cm^2)**	5.91	9.18	15.68	17.27	19.10
$\boldsymbol{R_{scl,min}}$ **(Ω cm^2)**	4.41	7.79	15.10	16.62	18.37

Supplementary Note 1. Heterodyne KPFM measurements of a thin-film battery with a grounded Au layer

For our heterodyne KPFM measurements on thin-film batteries, we normally use a configuration where the Au layer is connected to the working electrode of a potentiostat and the Li layer is connected to the counter electrode of a potentiostat. Additionally, the counter electrode is grounded. Here, the CPD measured by heterodyne KPFM of the LCO layer corresponds to the Volta potential (ψ). Changes in ψ are caused by a change of the electrochemical potential of electrons ($\tilde{\mu}_e$) in LCO or a change of the work function ($\Delta\Phi$) of LCO, represented by equation (1).

For comparison, we used a configuration where we reversed the electrical connection. Now, the Au layer is grounded and the counter electrode Li is connected to the working electrode of the potentiostat (Supplementary Figure 11 a). By grounding the Au layer, it fixes the electrochemical potential of electrons in LCO as well. In this configuration, the measured CPD changes on the LCO and Au layers result from work function differences between both materials. We want to point out, that KPFM directly measures the CPD between tip and sample, which is the difference between the work function of the sample region under the tip and the work function of the tip itself. When the grounding point of the battery is switched from anode to cathode, the entire electrochemical potential landscape of the battery shifts relative to the instrument's ground, which the tip is referenced to. Consequently, the absolute CPD values measured across the sample will change because the reference potential has been shifted. However, the scientifically relevant metric for analyzing the space charge layer is not the absolute CPD value, but the CPD change across the LPO|LCO interface.

Our measurements reveal that the CPD values of LCO and Au remain constant when changing the battery voltage (Supplementary Figure 11 b). A constant CPD indicates that the work function of LCO at distances greater than 50 nm from the LPO interface does not change with Li concentration inside the LCO (Supplementary Figure 11 b). In contrast, the CPD value of the LPO layer decreases with increasing battery voltage. In this measurement, the grounding of Au fixes the electrochemical potential of electrons in LCO. Thus, the fixed measured CPD in LCO layer indicates a constant work function of LCO at different battery voltages.

Supplementary Note 2. CPD measurements on unpolished surfaces

Breaking a thin-film battery results in a rough surface of the cross-section, which complicates finding the interface between the LPO and LCO layers in the topography measurements (Supplementary Figure 12 a – b). The heterodyne KPFM measurements on rough surfaces show that we can distinguish between the LPO and LCO layers (Supplementary Figure 12 c – e). However, the width of the transition region increases to around 100 – 150 nm (Supplementary Figure 12 f). We measured a width of 50 nm for polished cross-sections. Thus, a rough surface decreases the heterodyne KPFM lateral resolution.

We emphasize that the broken, but unpolished LPO and LCO surfaces show the same CPD value as polished surfaces for the same battery voltages. Furthermore, from the measured CPD change on the LPO and LCO layers for battery voltages of 3.0 V, 3.8 V and 4.2 V vs. Li/Li^+, we can calculate that the Volta potential of the LPO layer is almost constant while the Volta potential of the LCO layer increases with increasing battery voltage. Furthermore, the differences between the measured Volta potentials are equal to the differences in the battery voltage (Supplementary Figure 12 g). This phenomenon is the same for the polished cross-section of the thin-film battery, and it indicates that argon ion milling does not contribute to changes in the CPD values.

Supplementary note 3. DFT calculations of changes in LCO work function

The work functions of the (100) surface of Li_xCoO_2 (x=0.7, 0.8, 0.9 and 1) were computed using density functional theory (DFT).

We first employed a model where Li atoms are extracted from both the bulk and surface of Li_xCoO_2 (Supplementary Figure 13a). For this model, the work function of Li_xCoO_2 increases with decreasing x in Li_xCoO_2. Then we used a second model where Li atoms are extracted only from the bulk of Li_xCoO_2 (Supplementary Figure 13b). For this model, the work function of Li_xCoO_2 is almost constant and does not change with x in Li_xCoO_2. This means work function of LCO is mainly related to the Li content in the surface layer of Li_xCoO_2.

We further analyzed the work function change when Li atoms deintercalate from $LiCoO_2$ to reach the stoichiometry $Li_{0.7}CoO_2$. For $Li_{0.7}CoO_2$, we chose two models: in the first model Li atoms only deintercalate from the surface layer of $LiCoO_2$, while in the second model Li atoms only deintercalate from the bulk area of $LiCoO_2$. The results show that the work function of $LiCoO_2$ equals the work function of $Li_{0.7}CoO_2$ in the second model. On the other hand, the work function of $Li_{0.7}CoO_2$ in the first model increases by 0.87 eV (Supplementary Figure 14) compared to $LiCoO_2$.

Those above calculations confirm that only Li-ions extraction from the surface of $LiCoO_2$, the work function of $LiCoO_2$ can change. Therefore, in our thin-film battery the measured constant work function of LCO layer indicates that the Li content in the very surface of LCO exposed to the outside atmosphere remains constant, while it only changes within the bulk LCO area.

Supplementary note 4. Analyzing the contribution of the exchange of electrons/holes on the LPO | LCO interface space charge layer formation

The formation and evolution of the space charge layer at the LPO|LCO interface can be attributed to two principal mechanisms: (i) the transfer of electrons or holes between the two materials, or (ii) the redistribution of mobile Li ions across the interface to equilibrate the lithium chemical potential. To distinguish between these phenomena, we evaluated the electronic transfer hypothesis by analyzing the band alignment of the heterostructure and comparing the predicted outcome with our direct experimental observations.

We sketched the band structure diagram for Li, Li_3PO_4, $LiCoO_2$ (before contact), $LiCoO_2$ (at 2.0 V vs. Li/Li^+) and $Li_{0.5}CoO_2$ (at 4.3 V vs. Li/Li^+) in Supplementary Figure 16 based on our KPFM/NRA measurements results and reported values in literature[3-6].

For a newly fabricated thin-film battery, we measured an open circuit voltage of around 2.0 V vs. Li/Li^+. Now, $LiCoO_2$ is in direct contact with Li_3PO_4 and the band structure of $LiCoO_2$ (2.0 V vs Li/Li^+ state in Supplementary Figure 16) exhibits only a slightly higher Fermi level (E_f) and conduction band minimum (CBM) compared to the band structure of $LiCoO_2$ before contact[3-6]. This means that a space charge layer forms at the Li_3PO_4 | $LiCoO_2$ interface with positive charges at Li_3PO_4 and negative charges at $LiCoO_2$. This interpretation is consistent with the SXPS measurements (Figure 1, Supplementary Figure 2 and 3) carried out on $LiCoO_2$ surface characterization after Li_3PO_4 deposition. There are two possible causes of space charge formation. The first possibility is that electrons move from Li_3PO_4 to $LiCoO_2$. Before contact, the E_f and CBM of Li_3PO_4 are higher compared to $LiCoO_2$, then electrons have the tendency of moving from Li_3PO_4 to $LiCoO_2$ when they are in contact. The second possibility is that Li ions move from $LiCoO_2$ to Li_3PO_4 to maintain an equilibrium of the electrochemical potential of Li ions. At this point, we cannot determine which is the main reason for the space charge layer formation.

Continuously charging the thin-film battery increases its voltage state, which decreases E_f and the CBM of $LiCoO_2$ to 4.3 V vs Li/Li^+ as shown the band structure of $Li_{0.5}CoO_2$ (4.3 V state) in Supplementary Figure 16. This increases the thermodynamic driving force for electrons to transfer from LPO to LCO. If this electronic transfer were the dominant mechanism governing the space charge layer, one would expect an accumulation of negative charge (electrons) on the LCO side of the interface. Furthermore, this negative charge accumulation should become more pronounced with increasing battery voltage and increasing E_f difference becomes larger. However, this prediction is directly contradicted by our operando experimental results. The KPFM measurements reveal a potential increase on the LCO side of the interface during charging, indicating an accumulation of net positive charge. This finding is independently and quantitatively corroborated by our operando Nuclear Reaction Analysis (NRA) measurements, which directly track the lithium concentration and confirm a buildup of Li ions at the LCO interface.

The clear discrepancy between the predicted outcome of the electron-transfer model (negative charge accumulation) and our combined experimental observations from two independent techniques (positive charge accumulation) leads to the definitive conclusion that electronic redistribution is not the primary mechanism driving the SCL evolution. Instead, the data provides unambiguous evidence that the process is dominated by the redistribution of mobile Li ions. We propose that despite the thermodynamic driving force for electron transfer, the large band gap of LPO (~6 eV) introduces a significant kinetic barrier that impedes the movement of electrons from LPO to LCO, thus favoring the ionic redistribution pathway.

Supplementary note 5. Theoretical calculation of the Li ion concentration distribution at the LPO | LCO interface at different battery voltages

For our space charge layer calculation (basic parameters used for calculation are shown in Supplementary Table 1 – 2) an equilibrium voltage for LPO has not yet been reported, contrary to other solid electrolytes such as $Li_{1.2}Al_{0.2}Ti_{1.8}(PO_4)_3$ and $Li_{1.5}Al_{0.5}Ge_{1.5}(PO_3)_4$ with reported equilibrium voltages vs. Li/Li^+. Zhu Cheng *et al*. reported that the solid electrolyte equilibrium voltage vs. Li/Li^+ can be determined by measuring the voltage relaxation of a Li | solid electrolyte heterostructure device after external potential polarization[7]. Based on this, Issei Sugiyama *et al*.[8] reported that the voltage of a Li | LPO heterostructure cell relaxed and is stable at around 0.7 V vs. Li/Li^+ after being charged. Based on this measurement, we used an LPO equilibrium voltage of 0.7 V vs. Li/Li^+ in our space charge layer calculation as shown in Figure 4f.

Possibly, the LPO equilibrium voltage may be not accurate. Therefore, we also used 1.4 V, 2.1 V, 2.8 V and 3.5 V vs. Li/Li^+ as the LPO equilibrium voltages in our calculations as well. The equilibrium voltage of LPO cannot be higher than 3.8 V vs. Li/Li^+ because we have already observed a current in our thin-film battery after the voltage reached 3.8 V vs. Li/Li^+.

All the calculation results show the same space charge layer change tendency (Supplementary Figure 21) The Li-ion concentration on the LPO side in the space charge layer is smaller than in bulk LPO, while Li-ion concentration on the LCO side in the space charge layer is larger than in bulk LCO. Both differences in concentration increase with increasing battery voltage.

Supplementary Note 6. LPO | LCO interfacial R_{ct} calculation.

The reaction that takes place at the LCO | LPO interface is:

$$(1-x)Li^{+} + (1-x)e^{-} + Li_xCoO_2 \rightleftharpoons LiCoO_2 \tag{S1}$$

where *x* has a value between 0 and 1. The reaction rate of Li-ion insertion (r_i) and Li-ion extraction (r_e) from the LCO host can be expressed by[9-11]:

$$r_i = k_i\, c_{max}\, (1-x)\, [Li^{+}] \tag{S2}$$

$$r_e = k_e\, c_{max}\, x \tag{S3}$$

k_i is the insertion reaction rate constant, k_e is the extraction reaction rate constant, c_{max} is the maximum Li-ion insertion concentration in LCO and $[Li^{+}]$ is the bulk Li-ion concentration in LPO.

At a formal potential E^0, k_i^0 and k_e^0 are determined by the reaction activation energy according to the Arrhenius equation:

$$k_i^0 = P_i exp(-G_i^0/RT) \tag{S4}$$

$$k_e^0 = P_e \exp(-G_e^0/RT) \tag{S5}$$

P_i and P_e are pre-exponential factors for the Li-ion insertion and extraction reactions, respectively. G_i^0 and G_e^0 are the activation energies for Li-ion insertion and extraction reactions.

We use Frumkin's isotherm for describing Li-ion insertion into and extraction from LCO. Frumkin's isotherm considers the interaction between Li-ions and the LCO host[12]. Then we define the change of molar intercalation energy as ΔG, which results from Li-ion insertion into LCO. When a potential *E* is applied to the electrode, the activation energy of Li-ion insertion (G_i^E) and extraction reaction (G_e^E) can be written as:

$$G_i^E = G_i^0 - \alpha\, (FE + \Delta\mathrm{G}) \tag{S6}$$

$$G_e^E = G_e^0 + [(1-\alpha)\, (FE + \Delta\mathrm{G})] \tag{S7}$$

α is the transfer coefficient and *F* is the Faraday constant.

Then k_i the insertion reaction rate constant and k_e the extraction reaction rate constant change to:

$$k_i = k_i^0 \exp[\frac{-\alpha\, (FE+\Delta\mathrm{G})}{RT}] \tag{S8}$$

$$k_e = k_e^0 \exp[\frac{(1-\alpha)\, (FE+\Delta\mathrm{G})}{RT}] \tag{S9}$$

From equation S2, S3, S8 and S9, we can calculate the total reaction Faraday current *i* as:

$$i = F\, A\, c_{max}\, (1-x)\, k_i^0\ exp\left[\frac{-\alpha(FE+\Delta G)}{RT}\right]\, [Li^{+}] - F\, A\, c_{max}\, x\, k_e^0\, exp[\frac{(1-\alpha)\, (FE+\Delta G)}{RT}] \tag{S10}$$

A is the electroactive surface area.

In the equilibrium state, $E = E_e$ total current i is 0, the exchange current i_0 can be written as:

$$i_0 = F\ A\ c_{max}\ (1-x)\ k_i^0\ exp\left[\frac{-\alpha\ (FE_e+\Delta G)}{RT}\right]\ [Li^+] = F\ A\ c_{max}\ x\ k_e^0\ exp[\frac{(1-\alpha)\ (FE_e+\Delta G)}{RT}] \quad (S11)$$

Then

$$i_0 = F\ A\ c_{max}\ [Li^+]^{1-\alpha}\ (k_i^0)^{1-\alpha}\ (k_e^0)^{\alpha}\ (1-x)^{1-\alpha}\ x^{\alpha} \quad (S12)$$

Here, we define the standard reaction rate constant as k_0 at standard potential E_0,

$$k_0 = k_i^0\ \exp\left[\frac{-\alpha\ (FE_0+\Delta G)}{RT}\right] = k_e^0\ \exp[\frac{(1-\alpha)\ (FE_0+\Delta G)}{RT}] \quad (S13)$$

$$k_i^0 = k_0\ \exp\left[\frac{\alpha\ (FE_0+\Delta G)}{RT}\right] \quad (S14)$$

$$k_e^0 = k_0\ \exp\left[\frac{(\alpha-1)\ (FE_0+\Delta G)}{RT}\right] \quad (S15)$$

When we substitute equation S14 and S15 into equation S12,

$$i_0 = F\ A\ c_{max}\ [Li^+]^{1-\alpha}\ k_0\ (1-x)^{1-\alpha}\ x^{\alpha} \quad (S16)$$

From the definition of charge transfer resistance,

$$R_{ct} = \frac{RT}{Fi_0} \quad (S17)$$

Substituting i_0 in equation S17 with equation S13,

$$R_{ct} = \frac{RT}{F^2 A c_{\max} [Li^+]^{\alpha} k_i^0 (1-x)^{\alpha} x^{\alpha}}\, exp[\frac{\alpha(FE+\Delta G)}{RT}] \quad (S18)$$

The change of molar intercalation activation energy *ΔG*, which is related to the concentration of intercalated Li-ions in LCO, can be written as:

$$\Delta G = a + gx \quad (S19)$$

where *a* is a constant related with interaction between a intercalated Li-ion and a host LCO lattice near it, *g* is related to the interaction energy between two adjacent intercalated Li-ions in LCO[9].

Since Li-ion insertion and extraction from LCO is a reversible process, the transfer coefficient is α=0.5[13]. Then R_{ct} can be expressed as:

$$R_{ct} = \frac{RT}{F^2 A c_{\max} [Li^+]^{0.5} k_i^0 (1-x)^{0.5} x^{0.5}}\, exp[\frac{0.5(FE+a+gx)}{RT}] \quad (S20)$$

For Li_xCoO_2, in the voltage range around 3.8 V – 4.0 V vs. Li/Li^+, there is a plateau in the CV curve, this can be seen as a first-order phase transition[12,13]. In Frumkin's isotherm model, the *g* for a first-order phase transition has a value of 4.0[12-14]. In our calculations, we ignore kinetic effects resulting from space charge layer formation. Thus, we can use a *g* of 4.0 in the voltage range from 3.8 – 4.0 V vs. Li/Li^+.

When the voltage falls below 3.8 V vs. Li/Li^+, *x* in Li_xCoO_2 is almost constant compared with 3.8 V vs. Li/Li^+. In this case we use *g* equal to 4.0 as well. For a battery voltage higher than 4.1 V vs. Li/Li^+, *x* in Li_xCoO_2 decreases to a value smaller than 0.6. Then almost half of Li-ions are extracted from bulk. In this case, Frumkin's isotherm model is not valid, and we use Langmuir's isotherm model. In Langmuir's model, the interaction between two adjacent intercalated Li-ions is ignored. Thus, *g* equals 0[11].

To further shorten equation S20, we use n to replace the constant part $\frac{RT}{F^2 Ac_{\max}[Li^+]^{0.5}k_i^0} exp[\frac{0.5(FE+a)}{RT}]$. We get:

$$R_{ct} = \frac{n}{(1-x)^{0.5}x^{0.5}} \exp(0.5 * gx) \qquad \text{(S21)}$$

and g equals 4 when the battery voltage is lower than 4.0 V vs. Li/Li$^+$, while g equals 0 when the battery voltage is higher than 4.1 V vs. Li/Li$^+$.

Supplementary note 7. Determining the battery voltage when a space charge layer disappears at the LPO | LCO interface

A battery voltage of 4.2 V vs Li/Li$^+$ corresponds to a composition $Li_{0.55}CoO_2$[15-18]. Based on this composition reference, we calculated the Li content *x* in Li_xCoO_2 for different battery voltages from our charging-discharge curve (Figure 1f and Supplementary Table 1). For all values *x* in Li_xCoO_2 we calculated the LCO | LPO interfacial theoretical charge transfer resistance in dependence of battery voltage (Supplementary Table 3) by using equation 5 and equation S20. In our Supplementary Table 3, *n* is constant and corresponds to $\frac{RT}{F^2 A c_{max} [Li^+]^{0.5} k_i^0} exp[\frac{0.5(FE+a)}{RT}]$.

In our model thin-film battery, physical defects and interfacial side reactions can be ignored. Thus, we assume that the difference between the experimentally measured interfacial resistance and the theoretical interfacial charge transfer resistance is only due to a space charge layer resistance at the LPO | LCO interface. It is generally accepted that a space charge layer acts as an additional barrier for Li-ion movement across the interface. Therefore, we expect always a space charge layer resistance except for the specific battery voltage at which the space charge layer disappears.

Here, we define the difference between measured battery interfacial resistance and theoretical interfacial charge transfer resistance at the LPO | LCO interface as K, which is thought to be the space charger layer resistance (as shown in Supplementary Figure 25). For battery voltages below 3.6 V, the measured interfacial resistance is too high to be fitted. We only consider the voltage range from 3.6 V to 4.3 V vs. Li/Li$^+$, where the interfacial resistance can be measured and fitted. We assume that the voltage where the space charge layer disappears ($V_{no\ SCL}$) is at 3.6 V, 3.7 V, 3.8 V, 3.85 V, 3.9 V, 4.0 V, 4.1 V, 4.2 V and 4.3 V vs. Li/Li$^+$ respectively. For each assumption, we can calculate the corresponding K values at other battery voltages. For $V_{no\ SCL}$, it must meet the requirements that 1) K at $V_{no\ SCL}$ equals to 0, 2). From 3.6 V vs. Li/Li$^+$ to $V_{no\ SCL}$ K decreases with increasing battery voltage. 3) From $V_{no\ SCL}$ to 4.3 V vs. Li/Li$^+$, K increases with increasing battery voltage. 4) All K values should be positive.

Based on the above calculations, we find that only when $V_{no\ SCL}$ equals 3.85 V vs. Li/Li$^+$, all of the above-mentioned requirements can be met. Therefore, we determined that the voltage where the space charge layer disappears in the LPO|LCO interface should be around 3.85 V. In the voltage range from 3.6 V to 3.85 V vs. Li/Li$^+$, the interfacial resistance may not be accurate since the semicircles at mid- and low-frequency of the Nyquist plots do not fully form at this voltage range. Thus, we cannot exclude that $V_{no\ SCL}$ might be lower than 3.6 V vs. Li/Li$^+$. To further simplify the conclusion, we can conclude that $V_{no\ SCL}$ is $<$ 3.85 V vs. Li/Li$^+$. This assumption will simplify our next calculation and introduces a negligible error. We can generally assume that in the voltage range from 3.85 V to 4.3 V vs. Li/Li$^+$, the space charge layer resistance increases with increasing battery voltage.

Supplementary note 8. Calculation of the proportion of the space charge layer resistance relative to the interface resistance in a thin-film battery

We can calculate the LPO | LCO interfacial charge transfer resistance R_{ct} by equation 5 and S21 (more details in Supplementary Note 6). We define $\frac{e^{0.5*gx}}{(1-x)^{0.5}x^{0.5}}$ as $R_{ct\prime@U}$ when the Li content x in Li_xCoO_2, which corresponds to the charging state with a battery voltage of U ($U > 3.85$ V vs Li/Li^+). The LPO | LCO interface charge transfer resistance at U ($R_{ct@U}$) can be written as:

$$R_{ct@U} = R_{ct\prime@U} \cdot n \tag{S22}$$

Then we can calculate the ratio of the interfacial resistance between LPO | LCO at the voltage of 3.85 V ($R_{int@3.85V}$) and U ($R_{int@U}$) as $K_{int@U}$:

$$K_{int@U} = \frac{R_{int@3.85V}}{R_{int@U}} = \frac{R_{ct@3.85V} \cdot n + Y}{R_{ct@U} \cdot n + X} \tag{S23}$$

$R_{int@3.85V}$ and $R_{int@U}$ were calculated by fitting the EIS data at the corresponding battery voltage (Supplementary Table 4). The value X is the space charge layer resistance at a battery voltage U ($R_{scl@U}$). The value Y is the space charge layer resistance at 3.85 V vs. Li/Li^+ ($R_{scl@3.85V}$). We solve equation S23 as a function $Y(X)$:

$$Y = K_{int@U} \cdot X + K_{int@U} \cdot R_{ct\prime@U} \cdot n - R_{ct\prime@3.85V} \cdot n \tag{S24}$$

The space charge layer resistance in the LPO | LCO interface increases with increasing voltage in the range of 3.85 V – 4.3 V vs. Li/Li^+ (Supplementary Note 6). Therefore, Y must be smaller than X. We plot the function Y(X) (equation S24) in Cartesian coordinate system. To further decide the X range meets the requirement that Y must be smaller than X, we plot another function Y=X in the same Cartesian coordinate system. The X-axis value of the crosspoint of the two functions is the maximum value of $R_{scl@U}$. Also, in the voltage of 3.85 V – 4.3 V vs. Li/Li^+, $R_{scl@U}$ must be larger than 0 because the existence of space charge layer in this voltage range has been proven to bring additional resistance. Thus, Y and X in the function Y(X) must both be larger than 0. As a result, the crosspoint of function Y(X) and X-axis in Cartesian coordinate system is the minimum $R_{scl@U}$ at the LPO|LCO interface at voltage U.

Based on the above description, we plot function Y(X) at different battery voltage as shown in Supplementary Figure 26, we decide the maximum and minimum $R_{scl@U}$ as shown in Supplementary Table 5.

Our KPFM and NRA results further prove that $R_{scl@U}$ monotonically increase with U in the voltage range of 3.85 V to 4.3 V vs. Li/Li^+. However, the above calculations only show the maximum and minimum of $R_{scl@U}$ at voltage U under the condition that $R_{scl@U}$ is larger than $R_{scl@3.85V}$. When battery voltage is larger than 4.0 V vs. Li/Li^+, the difference between interfacial resistance and charge transfer resistance at the LPO | LCO interface has been shown to increase with battery voltage (Figure 6c and 6d). This means $R_{scl@U}$ must increase with battery voltage when battery voltage is higher than 4.0 V vs. Li/Li^+. Thus, the above calculations of maximum and minimum $R_{scl@U}$ at 4.1 V, 4.2 V and 4.3 V vs. Li/Li^+ meet the requirement that $R_{scl@U}$ monotonically increase with battery voltage. Therefore, the only remaining thing we need to do is to further calculate $R_{scl@4.0V}$ to make sure it is larger than $R_{scl@3.92V}$.

We use $R_{ct@3.93V}\ \cdot n$ to replace $R_{ct@3.85V}\ \cdot n$ in function Y(X) (equation S24). When U is 4.0 V,

$$Y = K_{int@4.0V} \cdot X + K_{int@4.0V} \cdot R_{ct'@4.0V}\ \cdot n - R_{ct'@3.93V}\ \cdot n \qquad \text{(S25)}$$

The new function in equation S25 is named Y_2(X). In the function, X is $R_{scl@4.0V}$ and Y is $R_{scl@3.93V}$. We plot the function Y_2(X) and Y=X in Cartesian coordinate system (Supplementary Figure S27). Then the X-axis value of the crosspoint of the two functions is the maximum value of $R_{scl@4.0V}$. Both $R_{scl@4.0V}$ and $R_{scl@3.93V}$ are positive value, the crosspoint of function Y_2(X) and X-axis in Cartesian coordinate system is the minimum $R_{scl@4.0V}$. The results show that calculated range of $R_{scl@4.0V}$ is larger than that calculated $R_{scl@4.0V}$ in Supplementary Table 5. Thus, Supplementary Table 5 shows the smallest range of space charge layer resistance at the LPO|LCO interface at different battery voltages 3.93 V, 4.0 V, 4.1V, 4.2 V and 4.3V vs. Li/Li^+.

Combining the above calculated $R_{scl@U}$, $R_{ct@U}$ and the measured $R_{int@U}$, we can get the exact maximum and minimum R_{scl} for different voltages at the LPO | LCO interface with a standard unit of Ω cm^2 with equation S26:

$$R_{scl@U} = R_{int@U}\ \cdot \frac{R_{scl@U}}{R_{ct'@U} \cdot n + R_{scl@U}} \qquad \text{(S26)}$$

The calculated $R_{scl@U}$ at battery voltage of 3.93 V, 4.0 V, 4.1 V, 4.2 V and 4.3 V vs Li/Li^+ with unit of Ω cm^2 are shown in Supplementary Table 6.

Supplementary references

1. de Klerk, N. J.; Wagemaker, M. Space-charge layers in all-solid-state batteries; important or negligible? *ACS Appl. Energy Mater.* **2018,** *1* (10), 5609-5618.
2. Sumita, M.; Tanaka, Y.; Ikeda, M.; Ohno, T. Theoretical insight into charging process in a Li_3PO_4 (100)/$LiFePO_4$ (010) coherent interface system. *Solid State Ion.* **2016,** *285*, 59-65.
3. Hikima, K.; Shimizu, K.; Kiuchi, H.; Hinuma, Y.; Suzuki, K.; Hirayama, M.; Matsubara, E.; Kanno, R. Operando analysis of electronic band structure in an all-solid-state thin-film battery. *Commun. Chem.* **2022,** *5* (1), 52.
4. Ensling, D.; Cherkashinin, G.; Schmid, S.; Bhuvaneswari, S.; Thissen, A.; Jaegermann, W. Nonrigid band behavior of the electronic structure of $LiCoO_2$ thin film during electrochemical Li deintercalation. *Chem. Mater.* **2014,** *26* (13), 3948-3956.
5. Ferber, T. H.; Cangaz, Ş.; Jaegermann, W.; Hausbrand, R. Interface reactivity of in-situ formed $LiCoO_2$-PEO solid-state interfaces investigated by X-ray photoelectron spectroscopy: Reaction products, energy level offsets and double layer formation. *Appl. Surf. Sci.* **2022,** *571*, 151218.
6. Lida, S.-i.; Terashima, M.; Mamiya, K.; Kimoto, T.; Sasaki, S. Improving the Stability of Li Metal Anode/Solid Electrolyte Interfaces via an Li_3PO_4 Intermediate Layer: An Investigation of Surface Chemistry and Electronic Band Structure. *J. Electrochem. Soc.* **2023,** *170* (9), 090503.
7. Cheng, Z.; Liu, M.; Ganapathy, S.; Li, C.; Li, Z.; Zhang, X.; He, P.; Zhou, H.; Wagemaker, M. Revealing the impact of space-charge layers on the Li-ion transport in all-solid-state batteries. *Joule* **2020,** *4* (6), 1311-1323.
8. Sugiyama, I.; Shimizu, R.; Suzuki, T.; Yamamoto, K.; Kawasoko, H.; Shiraki, S.; Hitosugi, T. A nonvolatile memory device with very low power consumption based on the switching of a standard electrode potential. *APL Mater.* **2017,** *5* (4), 046105.
9. Yongfang, L.; Haoquing, W. Theoretical treatment of kinetics of intercalation electrode reaction. *Electrochim. Acta* **1989,** *34* (2), 157-159.
10. Holzapfel, M.; Martinent, A.; Alloin, F.; Le Gorrec, B.; Yazami, R.; Montella, C. First lithiation and charge/discharge cycles of graphite materials, investigated by electrochemical impedance spectroscopy. *J Electroanal. Chem.* **2003,** *546*, 41-50.
11. Zhuang, Q.; Xu, J.; Fan, X.; Wei, G.; Dong, Q.; Jiang, Y.; Huang, L.; Sun, S. $LiCoO_2$ electrode/electrolyte interface of Li-ion batteries investigated by electrochemical impedance spectroscopy. *Sci. China, Ser. B: Chem.* **2007,** *50* (6), 776-783.
12. Levi, M.; Aurbach, D. Frumkin intercalation isotherm—a tool for the description of lithium insertion into host materials: a review. *Electrochim. Acta* **1999,** *45* (1-2), 167-185.
13. Vorotyntsev, M.; Badiali, J. Short-range electron-ion interaction effects in charging the electroactive polymer films. *Electrochim. Acta* **1994,** *39* (2), 289-306.
14. Levi, M. D.; Salitra, G.; Markovsky, B.; Teller, H.; Aurbach, D.; Heider, U.; Heider, L. Solid‐state electrochemical kinetics of Li-Ion intercalation into $Li_{1-x}CoO_2$: simultaneous application of electroanalytical techniques SSCV, PITT, and EIS. *J. Electrochem. Soc.* **1999,** *146* (4), 1279.
15. Ohzuku, T.; Ueda, A. Solid‐state redox reactions of $LiCoO_2$ (R3m) for 4 Volt secondary lithium cells. *J. Electrochem. Soc.* **1994,** *141* (11), 2972.
16. Dahéron, L.; Dedryvère, R.; Martinez, H.; Ménétrier, M.; Denage, C.; Delmas, C.; Gonbeau, D. Electron transfer mechanisms upon lithium deintercalation from $LiCoO_2$ to CoO_2 investigated by XPS. *Chem. Mater.* **2008,** *20* (2), 583-590.
17. Ohnishi, T.; Mitsuishi, K.; Takada, K. In Situ X-ray Diffraction of $LiCoO_2$ in Thin-Film Batteries under High-Voltage Charging. *ACS Appl. Energy Mater.* **2021,** *4* (12), 14372-14379.

18. Möller, S.; Satoh, T.; Ishii, Y.; Teßmer, B.; Guerdelli, R.; Kamiya, T.; Fujita, K.; Suzuki, K.; Kato, Y.; Wiemhöfer, H.-D. Absolute Local Quantification of Li as Function of State-of-Charge in All-Solid-State Li Batteries via 2D MeV Ion-Beam Analysis. *Batteries* **2021,** *7* (2), 41.
